# NAT10-mediated *N*^4^-acetylcytidine modification is required for meiosis entry and progression in male germ cells

**DOI:** 10.1093/nar/gkac594

**Published:** 2022-07-08

**Authors:** Lu Chen, Wen-Jing Wang, Qiang Liu, Yu-Ke Wu, Yun-Wen Wu, Yu Jiang, Xiu-Quan Liao, Fei Huang, Yang Li, Li Shen, Chao Yu, Song-Ying Zhang, Li-Ying Yan, Jie Qiao, Qian-Qian Sha, Heng-Yu Fan

**Affiliations:** MOE Key Laboratory for Biosystems Homeostasis, Life Sciences Institute, Zhejiang University, Hangzhou 310058, China; MOE Key Laboratory for Biosystems Homeostasis, Life Sciences Institute, Zhejiang University, Hangzhou 310058, China; Center for Reproductive Medicine, Department of Obstetrics and Gynecology, National Clinical Research Center for Obstetrics and Gynecology, Key Laboratory of Assisted Reproduction (Peking University), Ministry of Education, Beijing Key Laboratory of Reproductive Endocrinology and Assisted Reproductive Technology, Peking University Third Hospital, Beijing 100191, China; MOE Key Laboratory for Biosystems Homeostasis, Life Sciences Institute, Zhejiang University, Hangzhou 310058, China; MOE Key Laboratory for Biosystems Homeostasis, Life Sciences Institute, Zhejiang University, Hangzhou 310058, China; MOE Key Laboratory for Biosystems Homeostasis, Life Sciences Institute, Zhejiang University, Hangzhou 310058, China; Fertility Preservation Laboratory, Reproductive Medicine Center, Guangdong Second Provincial General Hospital, Guangzhou 510317, China; MOE Key Laboratory for Biosystems Homeostasis, Life Sciences Institute, Zhejiang University, Hangzhou 310058, China; MOE Key Laboratory for Biosystems Homeostasis, Life Sciences Institute, Zhejiang University, Hangzhou 310058, China; MOE Key Laboratory for Biosystems Homeostasis, Life Sciences Institute, Zhejiang University, Hangzhou 310058, China; Key Laboratory of Reproductive Dysfunction Management of Zhejiang Province, Sir Run Shaw Hospital, School of Medicine, Zhejiang University, Hangzhou 310016, China; College of Life Science, Zhejiang University, Hangzhou 310058, China; Key Laboratory of Reproductive Dysfunction Management of Zhejiang Province, Sir Run Shaw Hospital, School of Medicine, Zhejiang University, Hangzhou 310016, China; Center for Reproductive Medicine, Department of Obstetrics and Gynecology, National Clinical Research Center for Obstetrics and Gynecology, Key Laboratory of Assisted Reproduction (Peking University), Ministry of Education, Beijing Key Laboratory of Reproductive Endocrinology and Assisted Reproductive Technology, Peking University Third Hospital, Beijing 100191, China; Center for Reproductive Medicine, Department of Obstetrics and Gynecology, National Clinical Research Center for Obstetrics and Gynecology, Key Laboratory of Assisted Reproduction (Peking University), Ministry of Education, Beijing Key Laboratory of Reproductive Endocrinology and Assisted Reproductive Technology, Peking University Third Hospital, Beijing 100191, China; Fertility Preservation Laboratory, Reproductive Medicine Center, Guangdong Second Provincial General Hospital, Guangzhou 510317, China; MOE Key Laboratory for Biosystems Homeostasis, Life Sciences Institute, Zhejiang University, Hangzhou 310058, China; Key Laboratory of Reproductive Dysfunction Management of Zhejiang Province, Sir Run Shaw Hospital, School of Medicine, Zhejiang University, Hangzhou 310016, China

## Abstract

Post-transcriptional RNA modifications critically regulate various biological processes. *N*^4^-acetylcytidine (ac^4^C) is an epi-transcriptome, which is highly conserved in all species. However, the *in vivo* physiological functions and regulatory mechanisms of ac^4^C remain poorly understood, particularly in mammals. In this study, we demonstrate that the only known ac^4^C writer, *N*-acetyltransferase 10 (NAT10), plays an essential role in male reproduction. We identified the occurrence of ac^4^C in the mRNAs of mouse tissues and showed that ac^4^C undergoes dynamic changes during spermatogenesis. Germ cell-specific ablation of *Nat10* severely inhibits meiotic entry and leads to defects in homologous chromosome synapsis, meiotic recombination and repair of DNA double-strand breaks during meiosis. Transcriptomic profiling revealed dysregulation of functional genes in meiotic prophase I after *Nat10* deletion. These findings highlight the crucial physiological functions of ac^4^C modifications in male spermatogenesis and expand our understanding of its role in the regulation of specific physiological processes *in vivo*.

## INTRODUCTION

Rapid progress in uncovering and characterizing RNA modifications has been fueled by innovations in methodologies (1). To date, > 170 types of chemical RNA modifications have been reported (2), which are collectively termed the epi-transcriptome. The epi-transcriptome regulates various biological processes in nearly every aspect of the mRNA life cycle, including splicing, nuclear export, stability maintenance and turnover (3,4). There have been substantial efforts to prove that these modifications are extensively linked to developmental defects and diseases including cancer, mitochondrial diseases, neurological disorders and diabetes (5). However, owing to the limitations of sufficiently sensitive genome-wide mapping techniques and obstacles in sample collection, several studies have been conducted to explore the relevance and correlation of chemical modifications with the abundance of tRNAs and rRNAs in diseases (6,7). The most abundant and widely explored modification of mammalian mRNA is *N*^6^-methyladenosine (m^6^A) (8,9). This modification regulates a broad range of physiological functions related to reproduction and developmental disorders, viral infection, inflammation and various cancers (5,10,11). However, the underlying regulatory mechanisms and physiological consequences of other modifications, such as *N*^4^-acetylcytidine (ac^4^C) in mRNAs, remain poorly understood, especially in mammals.

*N* ^4^-acetylcytidine, a highly conserved RNA modification in eukaryotic and prokaryotic cells, was first identified in yeast [tRNA^Leu^ (12), tRNA^Ser^ (13,14)] and *Escherichia coli* (15–17. The deposition of ac^4^C on tRNAs promotes the fidelity of decoding (17–19 and maintains the thermotolerance of archaea (20). Subsequently, ac^4^C was detected on 18S rRNA in both humans and yeast, and was found to play a role in maintaining the accuracy of protein translation (21,22). Recently, ac^4^C has been characterized as a widespread marker of human mRNAs; it enhances transcript stability and translation efficiency (23). In contrast, a later study reported a different conclusion stating that ac^4^C sites are not directly detected in human and yeast mRNAs, but they can be induced via massive overexpression of acetyltransferase complexes (24). All documented ac^4^C events in tRNA, rRNA and mRNA are catalyzed by the only known ac^4^C writer, *N*-acetyltransferase 10 (NAT10) (in humans) or its homolog Kre33 (in yeast) (21,22). Moreover, the abundance of ac^4^C in human body fluids changes significantly under various disease conditions, suggesting that the occurrence of human diseases is highly related to ac^4^C (25). However, the potential pathogenic role of ac^4^C in disease and its physiological functions *in vivo* remain unclear and require further investigation.

Approximately one-third of couples worldwide are currently struggling with infertility problems; 50% of such cases are attributed to male infertility (26). However, the pathogenic mechanisms underlying male infertility have not been completely elucidated. Meiosis, the basis of sexual reproduction, is required to ensure genome stability and heritable diversity by generating haploid gametes through homologous pairing, synapsis, recombination and chromosome segregation (27). Homologous recombination, a hallmark of meiosis, is a critical driver of evolution via adaptation (28,29). Meiotic recombination starts with the formation of hundreds of programmed DNA double-strand breaks (DSBs) created by *Spo11* in ‘hotspot’ regions (30–33). Subsequently, the resected broken DNA ends are loaded with single-stranded DNA-binding proteins (RPA, DMC1 and RAD51) to facilitate homology recognition and strand invasion (32,34,35), thus promoting synapsis initiation by the assembly of the synaptonemal complex (SC) (36). Despite decades of research focusing on homologous synapsis and recombination, the mechanisms underlying the orchestration of these events remain poorly understood. Whether RNA modifications can shape these hallmark events at the transcriptional or post-transcriptional level remains to be established.

Here, we demonstrated that the expression of the only known ac^4^C writer, NAT10, has tissue and cell specificity—it is highly expressed in reproductive organs. We identified the occurrence of ac^4^C in mRNAs and showed that it is present at different levels in different organs and undergoes dynamic changes during spermatogenesis. Furthermore, we revealed that germ cell-specific inactivation of *Nat10* resulted in the inhibition of meiotic entry and defects in the synapsis of homologous chromosomes, meiotic recombination and repair of DNA DSBs during meiotic prophase I. These results highlighted the crucial physiological function of ac^4^C *in vivo* and expanded the repertoire of known epi-transcriptomic modifications.

## MATERIALS AND METHODS

### Animals

All the mice used in this study were maintained on a C57BL/6J genetic background. The *Stra8-GFPCre* knock-in mouse line was previously reported (37) and was kindly provided by Prof. Ming-Han Tong from the Shanghai Institute of Biochemistry and Cell Biology, Chinese Academy of Sciences. To construct germ cell-specific *Nat10* knockout mice, *Nat10*-floxed mice (*Nat10^flox/flox^*) were bred with the *Stra8-GFPCre* mouse line to excise *loxP*-flanked exons 4 and 5, thus generating *Nat10*-conditional knockout mice. The detailed gene targeting strategies are listed in Supplementary Figure S4A, and all primers used for polymerase chain reaction (PCR) genotyping are listed in Supplementary Table S2. All the mice used in this study were housed in the pathogen-free facility of the Laboratory Animal Center of Zhejiang University. The experimental procedures were approved by the Zhejiang University Institutional Animal Care and Research Committee (approval # ZJU20210252 to H.Y.F), and mouse care and use were conducted in accordance with the relevant guidelines and regulations of Zhejiang University.

### Histology and immunostaining

Testes and cauda epididymis extracted from wild type (WT) and *Nat10^fl/–^;Stra8-Cre* (*Nat10*-SKO) male mice were isolated immediately after cervical dislocation and fixed in Bouin's solution for 24 h at 4°C for histological analysis and in 4% paraformaldehyde (Sigma-Aldrich, 158127–500G) overnight at 4°C for immunostaining. After stepwise dehydration using an ethanol series, the samples were embedded in paraffin and sectioned using a Leica slicing machine (Leica Biosystems, Germany). The slides were deparaffinized, rehydrated and stained with hematoxylin and eosin (HE) for histological analysis. For immunofluorescence, after dewaxing and hydration, the sections were boiled in 10 mM sodium citrate buffer (pH 6.0) for 15 min, gradually cooled down to room temperature, washed in phosphate-buffered saline (PBS) with 0.1% Triton X-100 (PBST), blocked with 5% bovine serum albumin (BSA) for 1 h at room temperature and then incubated with primary antibodies overnight at 4°C. After washing three times with PBST, the sections were incubated with secondary antibodies and 4′,6-diamidino-2-phenylindole (DAPI) for 1 h at 25°C. The samples were then washed, mounted and analyzed using a fluorescence microscope (Carl Zeiss AG, LSM710, Germany). The antibodies used are listed in Supplementary Table S1.

### Whole-mount immunofluorescence

After removing the tunica albuginea from the testes, seminiferous tubules were gently dispersed. Untangled seminiferous tubules were then fixed with 4% paraformaldehyde and 0.5 mM CaCl_2_ in PBS overnight at 4°C. After washing with PBST, the seminiferous tubules were dehydrated using a graded ethanol series (25, 50, 75 and 100%) in PBST on ice for 1 h at each step. After rehydrating three times for 5 min (each time) in PBST, the seminiferous tubules were blocked in the blocking buffer (1% BSA and 4% donkey serum in PBST) for 1 h at room temperature and then incubated with primary antibodies at 4°C overnight. After washing in PBST, the tubules were incubated with secondary antibodies for 1 h at room temperature, washed with PBST, mounted and analyzed using a laser scanning confocal microscope (LSM710). The antibodies used for the whole-mount staining are listed in Supplementary Table S1.

### Spermatocyte nuclear spreading

Meiotic chromosome spreads from mouse spermatocytes were prepared according to a previously described protocol with some modifications (38). Specifically, we collected testes from 4-week-old male mice and removed the tunica albuginea. Seminiferous tubules were treated with a hypotonic buffer (30 mM Tris, 50 mM sucrose, 17 mM trisodium citrate dihydrate, 5 mM EDTA, 0.5 mM dithiothreitol and 0.5 mM phenylmethylsulfonyl fluoride; pH 8.2) for 40 min and then smashed in 60 μl of 100 mM sucrose buffer (pH 8.2). Fragmented testicular tubules were resuspended in 100 mM sucrose and dispersed into single cells. The suspension was then gently spread onto slides using 1% paraformaldehyde fixative buffer containing 0.15% Triton X-100 (pH 9.2). After 2 h of incubation in a humidity box at room temperature, the slides were thoroughly air-dried and washed three times in PBST before blocking. For immunostaining, seminiferous tubules were blocked with 5% BSA for 1 h at room temperature and incubated with primary antibody (Supplementary Table S1) at 4°C overnight. The slides were then washed and incubated with a secondary antibody. Laser confocal scanning images were captured using a confocal microscope (LSM710). Structured illumination microscopy (SIM) images were captured using a spinning disk confocal super-resolution microscope (Olympus, Xpore SPinSR). Semi-quantitative analysis of the fluorescence signals was conducted using the ImageJ software from the National Institutes of Health, USA.

### Analysis of mRNA expression using RT-qPCR (reverse transcription quantitative real-time PCR)

Total RNA from tissues and spermatocytes was reverse-transcribed using the PrimeScript II 1st strand cDNA synthesis kit (Takara, 6210A). A random primer (Takara, 3801) was used to guide reverse transcription. RT-qPCR was performed using *Power* SYBR^®^ Green PCR Master Mix (Thermo Fisher Scientific, 4367659) and an Applied Biosystems 7500 Real-Time PCR System (Thermo Fisher Scientific). Relative mRNA levels were normalized using endogenous *Gapdh*, and RT-qPCR experiments were performed using at least three independent replicates. All the primers used are listed in Supplementary Table S2.

### Analysis of protein expression using western blotting

Proteins from the testes and other tissues were extracted using a radioimmunoprecipitation assay buffer. Spermatogenic cells were lysed in β-mercaptoethanol-containing loading buffer and heated at 95°C for 10 min. Protein lysates (15–20 μg of total protein) were separated by sodium dodecyl sulfate (SDS)–polyacrylamide gel electrophoresis and transferred to polyvinylidene difluoride membranes. The membranes were then blocked with 5% non-fat milk in Tris-buffered saline containing 0.05% Tween-20 (TBST) at room temperature for 1 h. After probing with primary antibodies overnight at 4°C, the membranes were washed in TBST three times and incubated with horseradish peroxidase (HRP)-linked secondary antibody. Finally, bands in the membranes were detected using enhanced chemiluminescence (ECL) western blotting substrate (Thermo Fisher Scientific, 32106), and the intensities of the bands were quantitatively analyzed using ImageJ. The primary antibodies are listed in Supplementary Table S1, and all the unprocessed gel figures are shown in Supplementary Figure S11.

### Isolation of spermatogenic cells with FACS

Different types of spermatogenic cells (L-Z, P-D, MII and RS) were isolated from male mouse testes according to previously published methods (39). Briefly, after removing the tunica albuginea, the testes were incubated in 5 ml of Dulbecco's phosphate-buffered saline (DPBS) (Thermo Fisher Scientific, 14190144) containing 120 U/ml collagenase type I (Thermo Fisher Scientific, 17100017) at 32°C with gentle agitation for 10 min until the seminiferous tubules were dispersed. The dispersed seminiferous tubules were further digested with 5 ml of 0.25% trypsin (Gibco, 25200072) and 0.1 ml of 5 mg/ml DNase I (Sigma-Aldrich, DN25) at 32°C for 8 min. The digestion was terminated by adding 0.5 ml of fetal bovine serum (FBS) to inactivate trypsin. The cell suspension was filtered through a DPBS-pre-wetted 70 μm cell strainer (Corning, 352350) and centrifuged at 500 × *g* for 5 min at 4°C. The cell pellet was resuspended at a concentration of 10^6^ cells/ml in Dulbecco's modified Eagle's medium (DMEM) (Gibco, C11995500BT) with Hoechst 33342 (5 μg/10^6^ cells) (Thermo Fisher Scientific) and 5 μl of DNase I. The cell suspensions were incubated for 30 min at 32°C with gentle rotation and then centrifuged at 500 × *g* for 5 min at 4°C to remove the supernatant. The retained cells were stained with propidium iodide (1 μg/10^6^ cells; Sigma-Aldrich, 25535-16-4) at room temperature and filtered using a 40 μm cell strainer (Corning, 352340).

The cell populations were sorted based on their fluorescent intensity label with Hoechst 33342/propidium iodide staining by fluorescence-activated cell sorting (FACS) using a flow cytometer (BD Biosciences, FACS Aria II, USA). Hoechst 33342 (Thermo Fisher Scientific, H3570) was excited with a 355 nm UV laser, and the wide-emission spectrum of the dye was detected in two distinct channels: the ‘Hoechst Blue’ (DAPI, 450/20 band-pass filter) and the ‘Hoechst Red’ [Indo-1 (Blue), 670LP/610 LP band-pass filter].

### Enrichment of germ cells and somatic cells

Germ cells and somatic cells were enriched based on differences in adhesion ability, as previously described (40,41). Briefly, the first step was to prepare a single-cell suspension through a two-step enzymatic digestion using the FACS sample preparation method described in the above section; the testes without the tunica albuginea were incubated in 5 ml of DPBS containing 120 U/ml collagenase I at 32°C with gentle agitation for ∼8–10 min and then further digested with 5 ml of 0.25% trypsin and 0.1 ml of 5 mg/ml DNase I at 32°C for 8 min. The digestion solution was neutralized with FBS (0.5 ml), filtered through a 70 μm cell strainer and then centrifuged at 500 × *g* for 5 min at 4°C. The pellet was resuspended in 10 ml of ES cell culture medium (DMEM with l-glutamine, 15% FBS, P/S, Na pyruvate, NEAA and 0.1 mM 2-mercaptoethanol) and seeded in a 10 cm culture plate. Subsequently, the single-cell suspension was allowed to adhere and grow for 3 h at 37°C in a 5% CO_2_ atmosphere. The floating and weakly adherent cells were collected, transferred to a new 10 cm dish and cultured for another 2 h. After two rounds of adherence selection, the floating and weakly adhering cells were collected as germ cells and the attached cells on the dish were collected as somatic cells. These two fractions were filtered through a 70 μm cell strainer and centrifuged for 5 min at 4°C. Cell pellets were lysed with SDS or NucleoZOL reagent for subsequent western blotting and HPLC-MS/MS experiments.

### Total RNA isolation and poly(A)-RNA purification

Total RNAs from tissues and sorted spermatocytes were isolated using the NucleoZOL reagent (Macherey Nagel, 740404.200) and RNeasy Mini kit (Qiagen, 74106), respectively, according to the manufacturer's instructions, and the concentrations were quantified using Qubit (Thermo Fisher Scientific, USA). For high-performance liquid chromatography–tandem mass spectrometry (HPLC-MS/MS), polyadenylated RNA was isolated by two rounds of purification using the Dynabeads^®^ mRNA Purification Kit (Thermo Fisher Scientific, 61006) according to the manufacturer's protocol, and the purity of the isolated poly(A)-RNA was verified by RT-qPCR using specific primers for the detection of 18S rRNA and 28S rRNA.

### ac^4^C detection using HPLC-MS/MS

HPLC-MS/MS was conducted as previously described to determine the ac^4^C to C ratio in total RNA and mRNA (42,43). Briefly, 200–300 ng of total RNA or mRNA was treated with 1 U of nuclease P1 (Sigma-Aldrich, N8630) in 50 μl of buffer containing 100 mM ammonium acetate (pH 5.5; TCI, A2269), 2.5 mM NaCl and 0.25 mM ZnCl_2_ for 2 h at 37°C. This was followed by the addition of 3.5 μl of H_2_O, 6 μl of 10 × Antarctic phosphatase buffer (NEB, B0289S) and 0.5 μl of Antarctic phosphatase (NEB, M0289S) with an additional incubation at 37°C for 2 h. Following digestion, sample volumes were adjusted to 150 μl using distilled deionized water, and the samples were filtered using an Amicon Ultra-0.5 Centrifugal Filter Unit to remove enzymatic constituents (Millipore, UFC500396). After lyophilization, the samples were reconstituted in 50 μl of distilled deionized water (LC/MS grade) containing 20% acetonitrile (Thermo Fisher Scientific, A955-1), centrifuged three times at 12 000 × *g* for 15 min and 5 μl of the solution was injected into LC-MS/MS (SCIEX, QTRAP^®^ 6500^+^ LC-MS/MS, USA).

### Dot blot analysis of ac^4^C levels

The dot blot assay was performed as previously described with some modifications (44). Briefly, the indicated amounts of total RNA were denatured at 75°C for 5 min, which was followed by chilling on ice for 1 min. The RNA samples were loaded directly onto an Amersham Hybond-N^+^ membrane (GE Healthcare, RPN203B) and UV-cross-linked twice with a UV dose of 150 mJ/cm^2^. The membrane was blocked with 5% non-fat dry milk for 40 min at room temperature and incubated with anti-ac^4^C antibody (1:500 dilution) overnight at 4°C. After being washed three times and incubated with HRP-conjugated anti-rabbit IgG secondary antibody (Santa Cruz Biotechnology), the membrane was visualized using the ECL western blotting substrate (Thermo Fisher Scientific, 32106).

For internal standard detection, the membrane was incubated with 0.02% methylene blue (Sigma-Aldrich, M4159) in 0.3 M sodium acetate (pH 5.2) for 10–15 min and then washed with ddH_2_O. The relative signal density was quantified using the ImageJ software.

### RNA-seq

Three different stages of spermatogenic cells from WT and *Nat10*-SKO mice were collected for RNA-seq using the Smart-seq2 method, with minor modifications (45). Briefly, each sample with 10 μl of the original volume was lysed using 2 μl of lysis buffer [1% Triton X-100: RNase inhibitor = 4:1, including 0.35 μl of 1:1000 diluted External RNA Controls Consortium (ERCC) spike-in] and incubated with oligo(dT) primer and a deoxynucleoside triphosphate mixture at 72°C for 3 min, and Smart-seq2 reverse transcription reactions were performed to obtain cDNA. After the first-strand reaction, the cDNA was pre-amplified using a limited number of cycles (∼13 cycles). Sequencing libraries were constructed from 0.5 ng of cDNA using the TruePrep DNA Library Prep Kit V2 for Illumina (Vazyme, TD503) according to the manufacturer's instructions. Barcoded libraries were pooled and sequenced on the Illumina HiSeq X Ten platform in 151 bp paired-end mode.

### RNA-seq data analysis

Raw reads were trimmed and mapped to the mm10 genome as previously described (46). Uniquely mapped reads were employed to quantify gene expression using Htseq v0.11.2 and Cufflinks v2.2.1, and further normalized with the ERCC spike-in. The ERCC table was obtained as previously described (46) and further calibrated according to the amount of cell input. Differential gene expression analysis was conducted using the DESeq2 R package. An adjusted *P*-value of < 0.05 and fold change (FC) of *Nat10*-SKO/WT > 2 or < 0.5 (absolute log2 FC > 1) were used as statistical significances to identify differentially expressed genes (DEGs). Transcripts per million (TPMs) were calculated to estimate gene expression levels, normalized for gene length, and sequencing depth (47). The ERCC-calibrated counts and the TPMs are listed in Supplementary Table S3.

### Gene Ontology (GO) analysis

To explore the potential regulatory network between gene expression and ac^4^C modification, we obtained a list of genes shared between down-regulated genes and genes with decreased ac^4^C signals after NAT10 deletion, and we used FC > 2 or < 0.5 and *P*-value < 0.05 as the threshold to filter for significantly different expression levels. GO analysis was performed using the Database for Annotation, Visualization, and Integrated Discovery (DAVID) tool (https://david.ncifcrf.gov/tools.jsp) (48,49).

### Statistical analysis

Three replicates were performed for each experiment, and the data were presented as mean ± standard error of the mean (mean ± SEM). The results for the different groups were compared using a two-tailed unpaired Student's *t*-test. Statistical significance was presented as *P < 0.05, **P < 0.01, ***P < 0.001 and ****P < 0.0001; n.s. indicates *P* > 0.05.

## RESULTS

### NAT10 is highly expressed in reproductive organs and initiates dynamic changes during spermatogenesis

**Figure 1.**
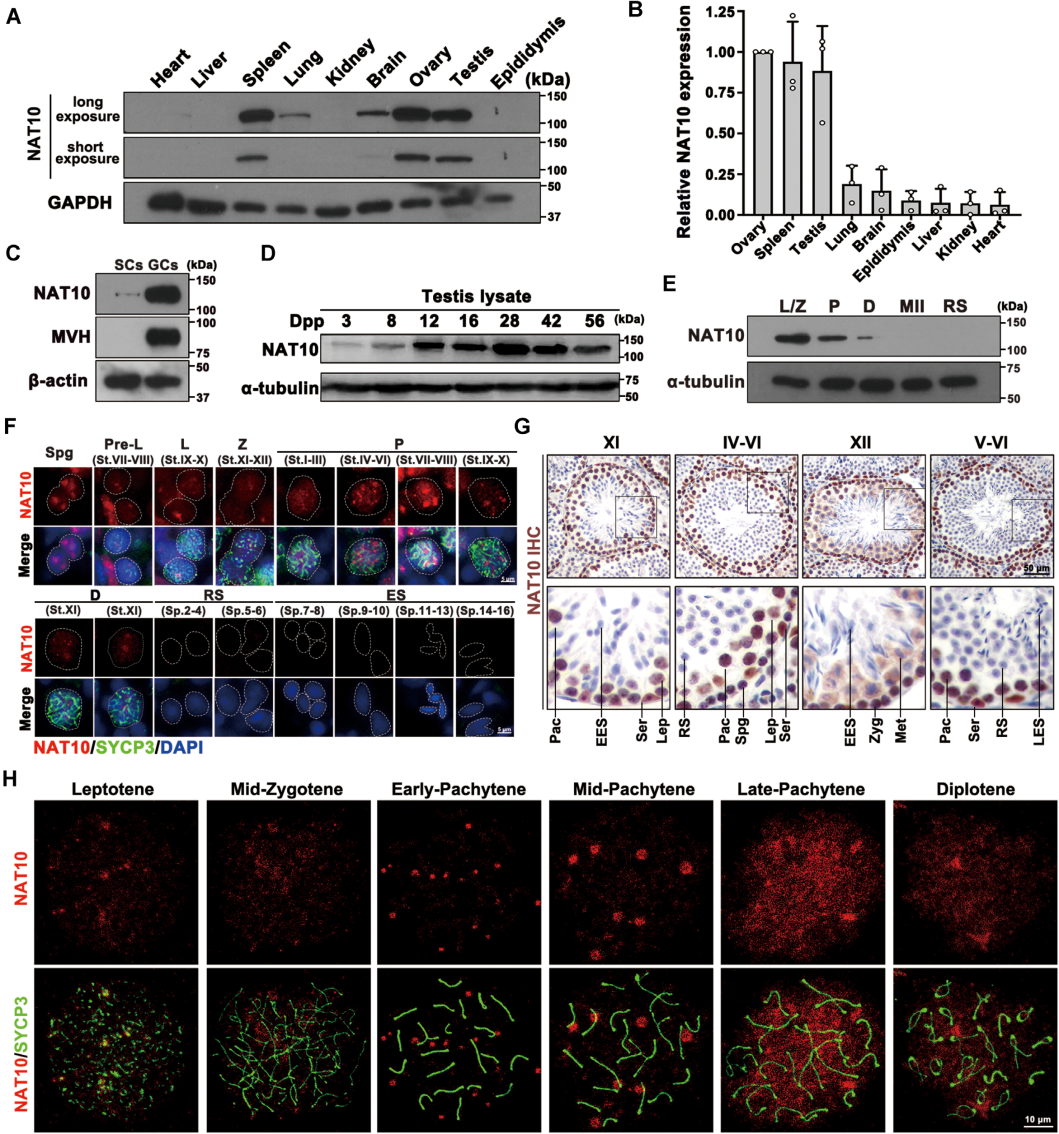
The expression of the ac4C writer NAT10 in multiple tissues and male germ cells. (**A**) Western blot analysis of NAT10 protein levels using anti-NAT10 and anti-GAPDH antibodies in the tissue lysates from 6-week-old WT mice. (**B**) Quantification of the relative expression levels of NAT10 in (A) using the ImageJ software. The relative expression level of the NAT10 protein was calculated by quantifying the gray value of NAT10/GAPDH for each sample. (**C**) Western blot analysis of NAT10 levels in the fraction of germ cells (GCs) and somatic cells (SCs) enriched from 6-week-old testes using a two-step enzymatic digestion process followed by a differential adhesion method. The GC marker MVH was used as an indicator of enrichment efficiency, and β-actin was used as the loading control. (**D**) Western blot analysis of NAT10 protein levels in mouse testes on different days postpartum (dpp) during the first wave of spermatogenesis. (**E**) Western blotting for NAT10 in spermatogenic cells isolated from adult WT mice using flow cytometry sorting (FACS) (L, leptotene; Z, zygotene; P, pachytene; D, diplotene; MII, metaphase II; RS, round spermatids). (**F**) Paraffin sections of WT adult testes were co-stained with rabbit anti-NAT10 and mouse anti-SYCP3 antibodies. The DNA was stained with DAPI (Spg, spermatogonia; PreL, pre-leptotene; L, leptotene; Z, zygotene; P, pachytene; D, diplotene spermatocytes; RS, round spermatids; ES, elongating spermatids). Scale bar = 5 μm. (**G**) Immunohistochemical staining of NAT10 in WT mouse testes (Spg, spermatogonia; Lep, leptotene; Zyg, zygotene; Pac, pachytene; Met, metaphase II; RS, round spermatid; EES, early elongating spermatids; LES, late elongating spermatids; Ser, Sertoli cells). Scale bar = 50 μm. (**H**) Localization of NAT10 (red) in spermatocytes at different stages of spermatogenesis in WT mice shown by nuclear spreading immunostaining. The meiotic stages of spermatocytes were determined by SYCP3 staining (green) of the chromosomal axis. Scale bar = 10 μm.

Sequence alignment and motif analyses revealed that NAT10 is a conserved protein expressed in multiple vertebrate species with a highly conserved *N*-acetyltransferase domain (616–654) and Glyc641, which was predicted to bind acetyl-CoA (50,51) (Supplementary Figure S1A–C). We first evaluated whether the expression of NAT10 was organ or cell specific. The results showed that the NAT10 protein was not ubiquitously expressed, but was enriched in the testes, ovaries and spleen (Figure 1A and B, and Supplementary Figure S2A).

Next, we explored the potential function of NAT10 and its mediated ac^4^C in mammalian spermatogenesis. Based on the discrepancy in the attachment ability of spermatocytes and Leydig cells, we used this differential adhesion method to enrich these two types of cells from 21 days postpartum (dpp) in testes (40,41). Western blotting results showed that NAT10 was more highly expressed in germ cells than in somatic cells (Figure 1C). The level of NAT10 protein increased gradually in the testes from postnatal day 12 to 28 and then gradually decreased during the first wave of spermatogenesis (Figure 1D). We performed flow cytometry sorting to isolate five developmental stages of mouse spermatogenic cells from WT adult male testes: leptotene/zygotene (L/Z), pachytene (P), diplotene (D) and metaphase II (MII) spermatocytes and round spermatids (RS). Western blotting results showed that NAT10 was expressed in the L/Z, P and D stages, with high enrichment in L/Z and P spermatocytes (Figure 1E). The extraction and reanalysis of published proteomic results (52) revealed that NAT10 is dynamically expressed during spermatogenesis and is enriched in meiotic prophase I spermatocytes (Supplementary Figure S2B and C). The localization and expression of NAT10 during spermatogenesis were further verified using immunohistochemistry and immunofluorescence staining. The results showed that NAT10 was expressed in spermatocytes in meiotic prophase I, spermatogonia and Sertoli cells (Figure 1F and G, and Supplementary Figure S2D). Surprisingly, chromosome spreading and immunofluorescence with two antibodies from different manufacturers showed that NAT10 exhibited dynamic pattern changes in diffused and condensed forms during meiotic prophase I (Figure 1H and Supplementary Figure S3A and B). We speculated that the dynamic change between the diffusion and aggregation of NAT10 may play an important physiological role.

### Dynamic ac^4^C modifications in tissues and mouse spermatogenesis

**Figure 2.**
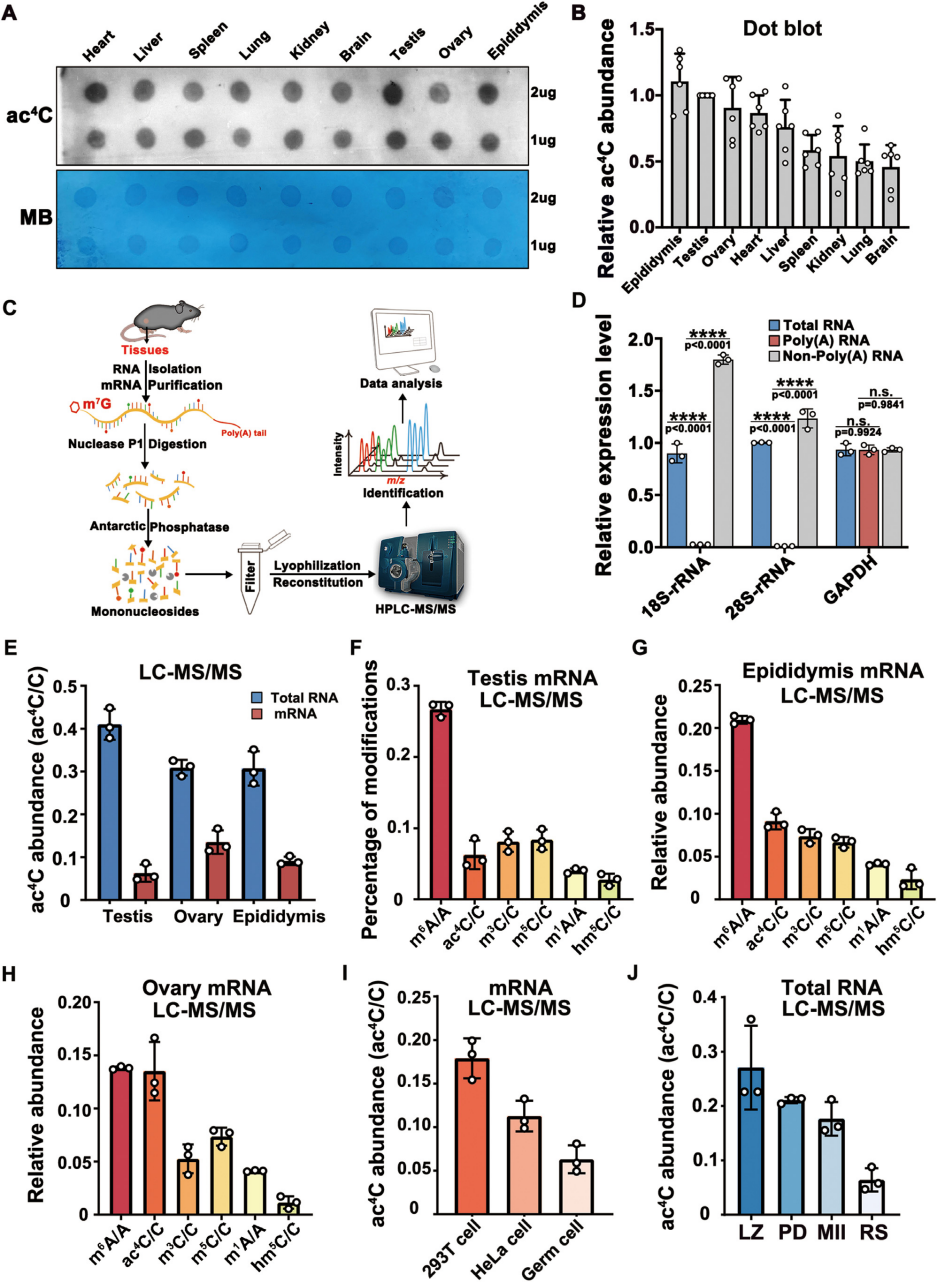
Dynamic ac^4^C modifications in tissues and mouse spermatogenesis. (**A**) Dot blot analysis of ac^4^C levels using the anti-ac^4^C antibody on total RNA from tissues of 6-week-old WT mice. Methylene blue staining was used as the internal standard. (**B**) Quantification of relative ac^4^C modification abundance in different samples using the ImageJ software; ac^4^C abundance was calculated by quantifying the gray value of ac^4^C/MB for each sample. Plots indicate the number of repetitions. (**C)** Schematic diagram of the HPLC-MS/MS experiment. (**D**) RT-qPCR detection of 18S and 28S rRNA expression to verify mRNA purity. Data are presented as the mean ± SEM; ****P < 0.0001. (**E**) LC-MS/MS detection of ac^4^C levels (ac^4^C/C) in total RNA and mRNA from WT adult testes, ovaries and epididymis. Data are presented as mean ± SEM, *n* = 3. (**F–H**) LC-MS/MS experiment to detect the abundance of m^6^A/A, ac^4^C/C, m^3^C/C, m^5^C/C, m^1^A/A and hm^5^C/C in the testes (F), epididymis (G) and ovaries (H). Data are presented as mean ± SEM, *n* = 3. (**I**) LC-MS/MS detection of ac^4^C levels in mRNA of 293T, HeLa and male germ cells. Mean ± SEM, *n* = 3. (**J**) LC-MS/MS detection of ac^4^C modification in spermatocytes at four different stages of spermatogenesis: L, leptotene; Z, zygotene; P, pachytene; D, diplotene spermatocytes; MII, metaphase II spermatocytes; RS, round spermatids. Data are presented as mean ± SEM of three biological replicates.

We performed dot blot to identify the overall abundance of ac^4^C modifications in total RNA samples from WT mouse tissues. Dot blot results showed that ac^4^C was present in the total RNA in all tissues (Figure 2A and B). Similarly, the abundance of ac^4^C modifications was higher in the total RNA of the epididymis, testes and ovaries than in other tissues (Figure 2A and B). Therefore, we selected the testis, epididymis and ovary to further detect the ac^4^C modification levels in both total RNA and mRNA samples using HPLC-MS/MS (Figure 2C). The mRNA was purified from total RNA using oligo(dT) beads, and enrichment efficiency was confirmed by detecting 18S and 28S rRNA levels via reverse transcription-quantitative real-time PCR (RT–qPCR) (Figure 2D). HPLC-MS/MS results showed that the abundance of ac^4^C mRNA was significantly lower than that of total RNA in the three tissues (Figure 2E). The abundance of ac^4^C modification in ovarian mRNA was ∼0.14%, followed by that of the epididymis and testis at 0.1% and 0.06%, respectively (Figure 2E).

A previous review summarized the comparison of the abundance of different chemical modifications based on the results of LC-MS/MS (53). Nonetheless, these results have been obtained by different research groups. To eliminate the errors caused by differences in samples, methods and equipment, we attempted to detect these chemical modifications simultaneously employing the same set of testicular total RNA and mRNA samples using HPLC-MS/MS. Our results showed that m^6^A modification of the mRNA was the most abundant in the testis, ovary and epididymis, while the abundances of other modifications were different in the three tissues (Figure 2F–H). Specifically, in the epididymis and testis, the abundance of ac^4^C modifications was lower than that of m^6^A (Figure 2F and G), especially in the testis; however, the ac^4^C modification level was almost equivalent to the m^6^A modification level in the ovary (Figure 2H). To compare the ac^4^C modification levels in different cell lines under the same instrument parameters, we used the differential attachment method to enrich testicular germ cells and simultaneously detected the ac^4^C content of mRNA from HeLa cells, 293T cells and germ cells via HPLC-MS/MS. Results showed that the percentages of ac^4^C modification on 293T cell mRNA reached 0.17%, while those on HeLa and germ cell mRNA were 0.11% and 0.06%, respectively (Figure 2I). Next, we investigated whether ac^4^C is dynamically regulated during spermatogenesis. We sorted four representative cell populations to detect ac^4^C modification. HPLC-MS/MS results showed that the abundance of ac^4^C modifications gradually decreased from the LZ stage to the RS stage, which was similar to the NAT10 protein expression pattern (Figure 2J). These results indicate that ac^4^C modification is widespread and that its abundance has tissue and cell specificity. In addition, ac^4^C modifications tend to decrease in abundance during spermatogenesis.

### NAT10 is essential for mouse spermatogenesis and male fertility

**Figure 3.**
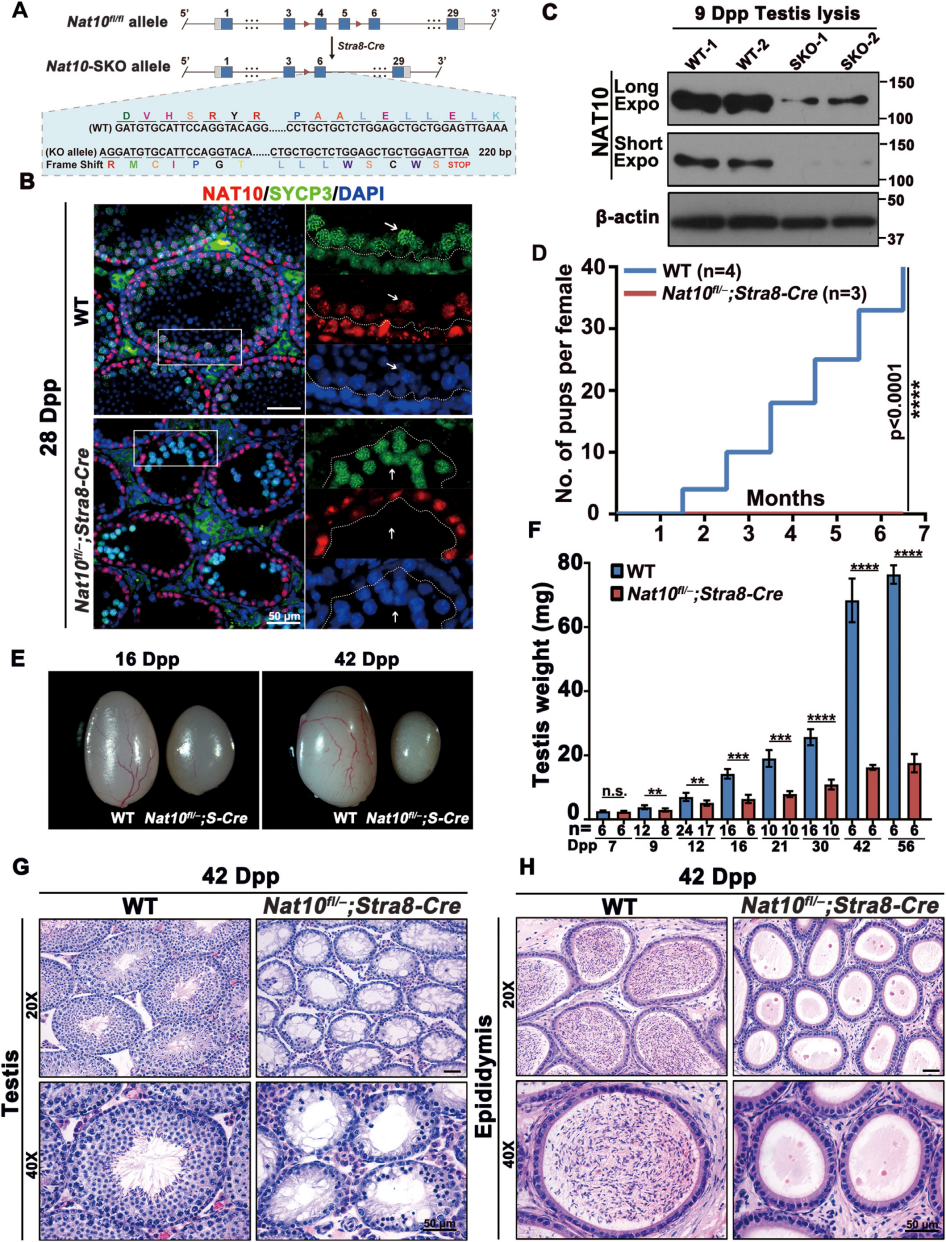
NAT10 is essential for mouse spermatogenesis and male fertility. (**A**) Schematic representation of the *Nat10* conditional targeting construct. Detailed information on the NAT10 isoforms and genotyping results are shown in Supplementary Figure S4A and B. (**B**) Immunofluorescence staining of NAT10 in WT and *Nat10*-SKO mouse testes. SYCP3 was co-stained to indicate spermatocytes. Scale bar = 10 μm. (**C**) Western blot analysis of NAT10 protein levels in WT control and *Nat10*-SKO testes. β-Actin was used as the loading control. (**D**) Fertility tests of *Nat10*-SKO and age-matched control mice for 6 months. Cumulative number of pups per male mating with one female mouse. ****P < 0.0001 via two-tailed Student's *t*-test. (**E**) Representative image showing the morphology of testes derived from *Nat10*-SKO and WT control mice at 16 dpp (left) and 42 dpp (right). (**F**) Weight of testes derived from WT and *Nat10*-SKO mice at the indicated ages. Error bars indicate SEM. **P < 0.01, ***P < 0.001 and ****P < 0.0001 by two-tailed Student's *t*-test. n.s. means not significant. (**G** and **H**) Morphological analysis of the testes (G) and epididymis (H) from control and *Nat10*-SKO mice using HE staining. Scale bar = 50 μm.

To study the physiological functions of NAT10 and its mediated ac^4^C modifications *in vivo*, we used *Stra8-GFPKI Cre* to cross with *Nat10*-floxed mice to obtain *Nat10^fl/–^;Stra8-GFP Cre* (hereafter referred to as *Nat10-*SKO) mice that specifically inactivated *Nat10* in germ cells before entering meiosis (Figure 3A and Supplementary Figure S4A and B). Immunofluorescence staining of sections and nuclear spreading from 4-week-old testes showed that NAT10 was expressed in SYCP3-positive cells in WT testes, but NAT10 signals were not detected in SYCP3-positive spermatocytes from the *Nat10-*SKO testes (Figure 3B and Supplementary Figure S4C). In addition, the expression of NAT10 in the 9-day-old testes lysate was detected using western blotting, and the results showed that NAT10 levels in the testes of *Nat10-*SKO mice were significantly lower than those in WT testes (Figure 3C), indicating that NAT10 was effectively deleted in *Nat10-*SKO mouse spermatocytes.

Subsequently, to verify whether the ablation of NAT10 has an impact on mouse reproduction, we mated control male mice (*Nat10^fl/+^* and *Nat10^fl/fl^* littermate males) and *Nat10-*SKO mice with WT female mice. The results of the fertility test for > 6 months showed that the *Nat10-*SKO male mice were completely infertile (Figure 3D). The testicular morphology and weight of *Nat10-SKO* mice 7 days after birth did not differ from those of the control mice. However, with an increase in age, the testis weight of *Nat10-*SKO mice was significantly lower than that of control mice (Figure 3E and F). Compared with the WT, there was no mature sperm in the epididymis, and the number of germ cells in the seminiferous tubules was significantly reduced in *Nat10*-SKO mice (Figure 3G and H). These results illustrate that NAT10 and its mediated ac^4^C are required for mouse testis development and male fertility.

### NAT10 is crucial for meiotic entry and spermatogonial differentiation

**Figure 4.**
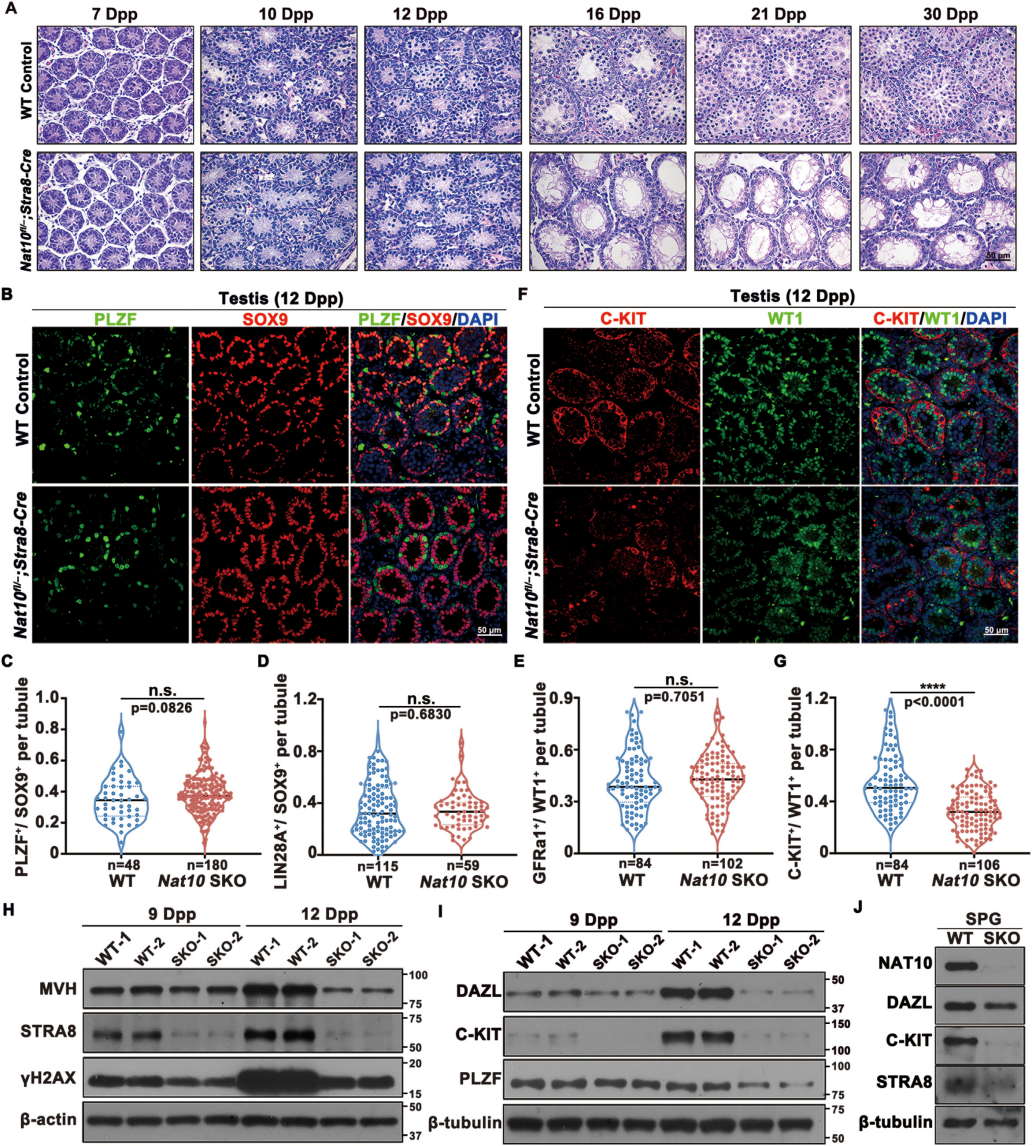
NAT10 is crucial for meiotic entry and spermatogonial differentiation. (**A**) HE staining of control and *Nat10*-SKO testes at the indicated ages. Scale bar = 50 μm. (**B**) Immunofluorescence co-staining for the undifferentiated spermatogonia marker PLZF (green) and Sertoli cell marker SOX9 (red) in the control and *Nat10*-SKO testes at 12 dpp. Scale bar = 50 μm. (**C**) Quantification of the ratio of PLZF-positive spermatogonia/SOX9-positive cells per tubule in histological sections of the control and *Nat10*-SKO 12 dpp testes. n.s. indicates not significant. (**D** and **E**) Quantification of the ratio of LIN28A-positive spermatogonia/SOX9-positive cells and GFRα1-positive spermatogonia/WT1-positive cells per tubule in histological sections of the control and *Nat10*-SKO 12 dpp testes relative to those shown in Supplementary Figure S6A and S6B. n.s. means not significant. (**F** and **G**) Immunostaining (F) and quantification (G) of c-KIT^+^ cells/WT1^+^ cells per tubule in sections from the 12 dpp control and *Nat10*-SKO mice. ****P < 0.0001 via two-tailed Student's *t*-test. (**H**) The expression levels of representative proteins in meiotic entry were significantly reduced after *Nat10* deletion in 9 and 12 dpp testes. (**I**) Western blot detection of the levels of key proteins that function in spermatogonial differentiation in the 9 and 12 dpp testes. (**J**) Western blot detection of key protein levels in isolated SPG (spermatogonial cells).

To trace the specific time-point when the number of germ cells began to decrease in *Nat10-*SKO male mice, we collected testicular samples at 7, 10, 12, 16, 21 and 30 dpp for HE staining (Figure 4A). The results of the morphological analysis showed that there was no difference between *Nat10-*SKO and the control at 7 dpp. Twelve days after birth, the number of germ cells in the seminiferous tubules began to decrease in the *Nat10-*SKO mice, followed by a sharp decrease in the testes from 16 to 30 dpp (Figure 4A). To verify whether the depletion of NAT10 affects meiotic entry, we used immunofluorescence staining to detect STRA8 (a marker for differentiating spermatogonia and pre-leptotene spermatocytes) and SYCP3 (a marker of meiotic spermatocytes) at 10, 12, 16 dpp and in adult testes (Supplementary Figure S5A–F). Quantitative statistics showed that the proportions of STRA8^+^ and SYCP3^+^ tubules in *Nat10-*SKO mice were significantly reduced (Supplementary Figure S5A–F). Western blotting was used to further detect the expression levels of key proteins involved in meiosis, such as MVH, STRA8 and γH2AX, and we found that the expression levels of all these proteins were significantly reduced in *Nat10-*SKO mice (Figure 4H), thus indicating that NAT10 plays an important role in meiotic entry. Furthermore, we detected apoptosis via the terminal deoxynucleotidyl transferase (TdT) dUTP nick-end labeling assay, and the results showed that a large number of apoptotic cells had already appeared in *Nat10-*SKO mice at 12 dpp (Supplementary Figure S5G and H).

Next, we examined spermatogonial differentiation in *Nat10-*SKO and control testes. Immunofluorescence staining detected undifferentiated spermatogonia markers such as PLZF, LIN28A and GFRα1, which were co-stained with SOX9 or WT1 to label Sertoli cells, and the ratio of undifferentiated spermatogonia to Sertoli cells at 12 days after birth and in adult testes was quantified. The results showed that the ratio of undifferentiated spermatogonia to Sertoli cells did not change (Figure 4B–E and Supplementary Figure S6A–B). Subsequently, whole-mount staining of seminiferous tubules for LIN28A and GFRA1 revealed consistent results (Supplementary Figure S6C), suggesting that the formation of the undifferentiated spermatogonial pool was not affected by NAT10 deletion. Next, we focused on the stages of spermatogonial differentiation using the differentiated spermatogonial cell marker, c-KIT. The ratio of KIT-positive cells to Sertoli cells significantly decreased in the seminiferous tubules of *Nat10-*SKO mice (Figure 4F and G, and Supplementary Figure S6D). We also detected these markers in adult testes, which showed similar results in that the undifferentiated spermatogonial pool was not affected (Supplementary Figure S6E–H), but the differentiated spermatogonial pool was reduced after *Nat10* deletion (Supplementary Figure S6I and J). DAZL and c-KIT proteins were also significantly reduced in both testes and isolated spermatogonia after *Nat10* deletion (Figure 4I and J). Collectively, these results indicated that *Nat10* deletion leads to defects in spermatogonial differentiation and meiotic entry.

### NAT10 deficiency resulted in defects during synapsis and meiotic recombination

**Figure 5.**
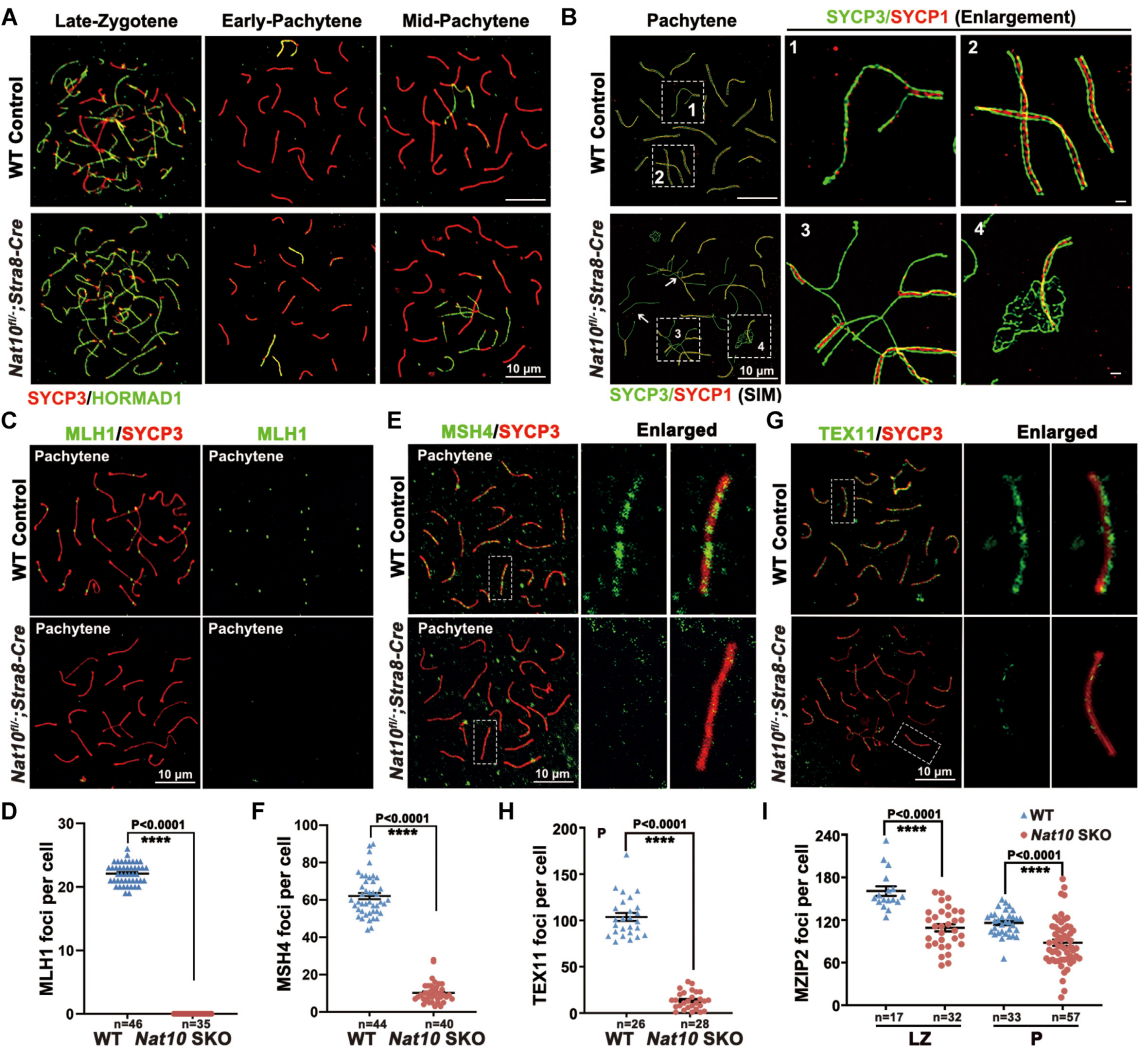
NAT10 depletion causes defects in synapsis and meiotic recombination. (**A**) Co-immunofluorescence staining of HORMAD1 and SYCP3 on surface-spread spermatocytes from the control and *Nat10*-SKO mouse testes. Scale bar = 10 μm. (**B**) Representative SIM images from super-resolution microscopy of the immunostaining of spread pachytene spermatocytes with SYCP1 and SYCP3 in WT and *Nat10*-SKO mice. The enlarged images highlight the detailed structure of the lateral and central axes. Scale bar = 10 μm. (**C** and **D**) The number of crossovers marked by MLH1 foci was significantly reduced in *Nat10*-SKO pachytene spermatocytes compared with that in WT pachytene spermatocytes (C). Quantification of MLH1 foci in WT and *Nat10*-SKO spermatocytes at pachytene stage (D). ****P < 0.0001 by two-tailed Student's *t*-test. Scale bar = 10 μm. (**E** and **F**) Pachytene-stage spermatocytes co-stained for SYCP3 and MSH4, with magnified views enlarged on the right (E). Quantification of MSH4 foci on nuclear surface spreads of WT and *Nat10*-SKO spermatocytes at the pachytene stage (F). ****P < 0.0001 via two-tailed Student's *t*-test. (**G** and **H**) Immunostaining (G) and quantification (H) of TEX11 in WT and *Nat10*-SKO pachytene spermatocytes. ****P < 0.0001 via two-tailed Student's *t*-test. Scale bar = 10 μm. (**I**) Quantification of number of MZIP2 foci in late zygotene and pachytene spermatocytes relative to those in Supplementary Figure S7D. ****P < 0.0001.

In the above investigation, we observed that *Nat10-*SKO mice contained spermatocytes that could enter meiosis. Thus, we further analyzed the proportion of haploid, diploid and tetraploid spermatocytes in both *Nat10-*SKO and WT testes using FACS. The results showed that haploid RS could not be detected in *Nat10*-SKO testes, and the number of tetraploid spermatocytes decreased sharply (Supplementary Figure S7A and B). We explored whether NAT10-mediated ac^4^C modification plays a role in homologous pairing, synapsis, recombination and chromosome segregation. First, we used nuclear spreading of spermatocytes coupled with immunofluorescence staining to detect the lateral element SYCP3 and central element SYCP1 of the SC. The results showed that synapsis was completed in autosomal axes at mid-pachynema of meiosis I in WT spermatocytes (abnormal synapsis: 6.2 ± 1.72%, *n* = 632). However, there were still non-synaptic homologous chromosomes in *Nat10-*SKO pachytene-stage spermatocytes (abnormal synapsis: 77.4 ± 5.17%, *n* = 580) (Supplementary Figure S7C). In pachynema, HORMAD1 was removed from the autosomal axes and retained only in the unpaired regions of the XY body, whereas NAT10-deleted spermatocytes retained HORMAD1 signals in several incomplete synaptic chromosomes (Figure 5A).

To precisely observe subcellular localization and synaptonemal complex structures, we used super-resolution SIM to investigate the dynamic architecture of the SC. Autosomes were completely paired in WT pachytene cells (Figure 5B). However, after NAT10 deletion, the chromosomes in most spermatocytes were not completely matched (Figure 5B). Further analysis led to the classification of synaptic defects into four types: the first was completely unpaired, the second had partial pairing and exhibited zipper-like chromosome forks and the third was the formation of a multichromosome structure, in which non-homologous chromosomes were paired (Figure 5B-3). The fourth type had fragmented ends of synaptic complexes and formed abnormal aggregates (Figure 5B-4). These results indicate that NAT10 is essential for homologous chromosome pairing and synapsis during meiotic prophase I.

Next, we evaluated whether NAT10 deletion affects recombination. Nuclear spreading and immunofluorescence results showed that MLH1 foci could not be detected in *Nat10*-SKO pachytene spermatocytes (Figure 5C and D), indicating that NAT10 deletion causes serious defects in crossover formation. The deletion of NAT10 also severely abolished the localization of MSH4 and TEX11 (two components of ZMM proteins) to the chromosomal axes in the pachytene stage (Figure 5E–H), suggesting that NAT10 is required for the precise localization of ZMM proteins. Recently, it was reported that MZIP2 facilitates the assembly of ZMM foci and promotes crossover (54). Immunostaining for MZIP2 showed that the number of MZIP2 foci was reduced in both leptonema and zygonema after *Nat10* deletion (Figure 5I and Supplementary Figure S7D). These results demonstrated that NAT10 is essential for meiotic recombination.

### NAT10 is critical for DSB repair in meiotic prophase I

**Figure 6.**
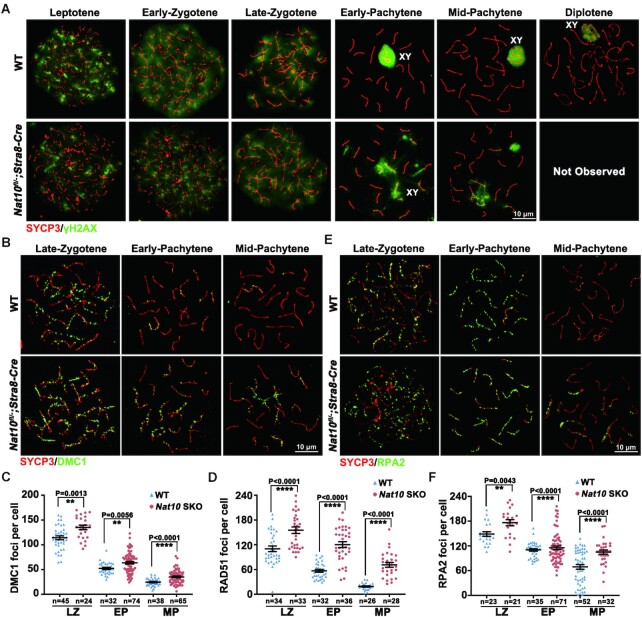
NAT10 depletion causes defects in DSB repair. (**A**) Representative images of WT and *Nat10*-SKO spermatocytes at different stages of meiotic prophase immunostained for SYCP3 and γH2AX. ‘Not observed’ means that spermatocytes in the diplotene stage were not observed in nuclear spreading. Scale bars = 10 μm. (**B** and **C**) Chromosome spreads of spermatocytes (LZ, late zygotene; EP, early pachytene; MP, mid-pachytene) from the testes of WT and *Nat10*-SKO males immunostained for DMC1 and SYCP3. (B). Quantification of DMC1 foci number in the indicated spermatocytes. Error bars indicate SEM. **P < 0.01 and ****P < 0.0001 via two-tailed Student's *t*-test (C). (**D**) Quantification of RAD51 foci number in zygotene and pachytene spermatocytes relative to those in Supplementary Figure S7E. ****P < 0.0001. (**E** and **F**) RPA2 and SYCP3 were detected in the nuclear surface spreads of WT and *Nat10*-SKO spermatocytes (E). Quantification of RPA2 foci numbers in the indicated spermatocytes. **P < 0.01 and ****P < 0.0001 via a two-tailed Student's *t*-test (F).

In WT spermatocytes, signals of γH2AX, a marker of DNA DSBs, were observed in most chromosomes during leptonema and zygonema stages. In the mid-late pachynema and diplonema, DSBs were repaired, and the γH2AX signal disappeared from autosomes and remained only on non-synapsed sex chromosomes (unrepaired γH2AX in autosomes: 2.2 ± 4.44%, *n* = 555) (Figure 6A). In *Nat10-*SKO spermatocytes, there was no significant difference in the γH2AX signal between leptonema and zygonema. However, we observed an expansion of the γH2AX signal from sex chromosomes to autosomes in pachytene spermatocytes (unrepaired γH2AX in autosomes: 72.2 ± 21.05%, *n* = 730) (Figure 6A), indicating defects in DSB repair.

To further investigate NAT10 function in the repair of DSBs, we examined the recombinase foci, Immunostaining and quantification of these two DSB repair markers showed decreased numbers of DMC1 and RAD51 foci with the transition from the leptotene to pachytene stage in WT spermatocytes, indicating the successful repair of DSBs. However, many DMC1 (Figure 6B and C) and RAD51 (Figure 6D and Supplementary Figure S7E) foci were retained in both late zygotene and pachytene stages of *Nat10-*SKO spermatocytes. Furthermore, we characterized the single-stranded DNA-binding protein RPA2; the number of RPA2 foci decreased at early/mid-pachytene in WT spermatocytes but was still maintained at a relatively high level in *Nat10*-SKO spermatocytes (Figure 6E and F). Western blotting results further confirmed that DMC1 and RPA2 levels were accumulated after *Nat10* deletion (Supplementary Figure S7F). These results suggest that the repair of DSBs is disrupted in the absence of NAT10.

### Loss of *Nat10* causes transcriptional dysregulation in mice

To gain insights into the molecular function of NAT10 in spermatogenesis, we isolated spermatogenic cells, including spermatogonia (SPG), pre-leptotene (PreL) and LZ stages, using FACS. After verifying the purity of the sorted cells (Supplementary Figure S8A and B), we used Smart-seq2 to construct the library and perform high-throughput transcriptome sequencing. Gene expression levels were assessed as TPM mapped reads, and the relative mRNA copy number between different samples was evaluated using the ERCC spike-in. All samples were analyzed at least in duplicate, and there was a high correlation between duplicates (Supplementary Figure S9A and Supplementary Table S4).

**Figure 7.**
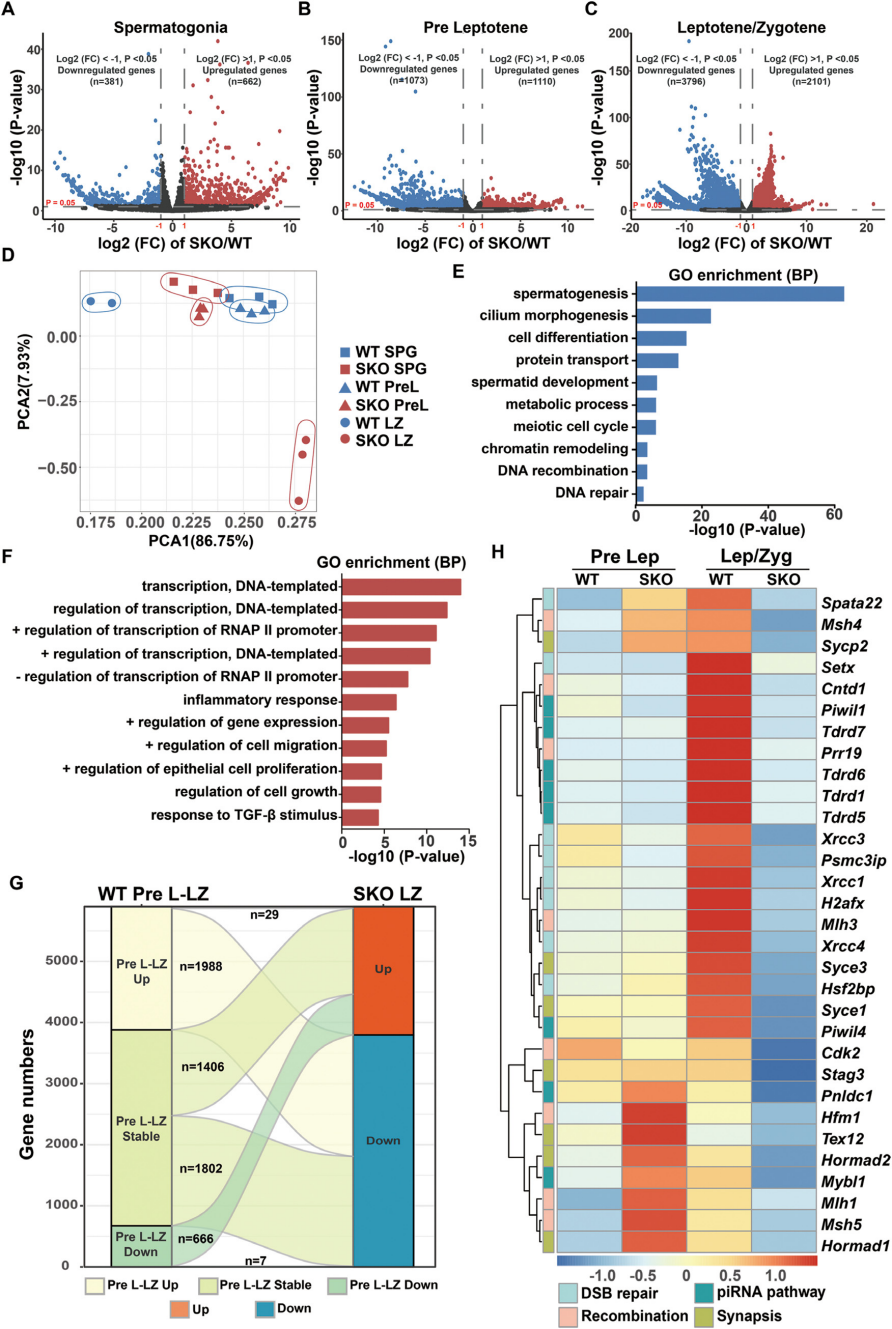
Loss of NAT10 causes transcriptional dysregulation. (**A–C**) Volcano plots show the number of significantly differentially expressed genes (DEGs) in spermatogonia (A), pre-leptotene (B) and leptotene/zygotene (L/Z) (C) that were isolated with FACS from WT and *Nat10*-SKO testes. Up-regulated and down-regulated genes are highlighted by red and blue dots, respectively. Those that are not DEGs are represented as dark gray dots. *P* threshold ( = 0.05) and log_2_FC threshold ( = ±1) are reported in gray horizontal and vertical dashed lines, respectively. n, gene number; FC, fold change. (**D**) PCA results of spermatogonia and pre-leptotene and leptotene/zygotene (L/Z) in WT and *Nat10*-SKO mice. Each symbol represents an RNA-seq sample, and WT and *Nat10*-SKO samples are shown in blue and red, respectively. Sample groups with similar gene expression profiles were clustered with the indicated colors. The proportions of variation in PCA1 and PCA2 were 86.75% and 7.93%, respectively. (**E**) GO enrichment analysis of biological processes (BP) indicates the potential functions of the down-regulated transcripts in the leptotene/zygotene stage derived from *Nat10*-SKO and WT mice (adjusted *P* < 0.05, FC > 2). (**F**) GO analysis indicates the potential functions of transcripts up-regulated by > 2-fold in leptotene/zygotene (adjusted *P* < 0.05, FC > 2). (**G**) Sankey diagram showing the expression pattern of transcripts at the pre-leptotene and leptotene/zygotene stages between WT and *Nat10*-SKO mice. Each rectangle represents a gene category. (**H**) Heatmap for four major functional categories of selected key genes showing distinct expression characteristics between pre-leptotene and leptotene/zygotene cells of WT and *Nat10*-SKO mice. The color key from red to blue indicates the relative gene expression levels from high to low.

Transcriptome profiling results showed that the loss of *Nat10* in the SPG stage resulted in down-regulation of 381 genes and up-regulation of 662 genes, respectively (Figure 7A). In addition, the numbers of down-regulated and up-regulated genes during the pre-leptotene period were 1073 and 1110, respectively (Figure 7B and Supplementary Table S5). GO analysis of the down-regulated genes after NAT10 deletion in the SPG and PreL stages showed that some genes were enriched in spermatogenesis and cell cycle processes (Supplementary Figure S9B and C and Supplementary Table S6). However, deletion of NAT10 resulted in a significant decrease in the expression levels of 3796 transcripts and an increase in the expression levels of 2101 transcripts in the leptotene/zygotene stage (Figure 7C and Supplementary Table S5). Furthermore,principal component analysis (PCA) showed that the components of the WT and SKO samples were slightly different in both the SPG and PreL stages, while the distribution of the LZ-stage sample diverged (Figure 7D), suggesting that the transcripts of the WT and *Nat10*-SKO were significantly different. Therefore, we focused on the LZ stage to explore the effect of NAT10 deletion on transcript turnover. GO analysis of the 3796 genes down-regulated in LZ revealed that clustering occurred during spermatogenesis, protein transport, homologous recombination and DNA repair (Figure 7E, Supplementary Figure S9D and Supplementary Table S6), which is consistent with previously observed phenotypes (Figures 3–6). In addition, GO analysis of the up-regulated genes in the LZ did not show significant biological process enrichment; most of them were enriched in the transcriptional regulation process (Figure 7F and Supplementary Table S6). We speculate that these up-regulated genes were activated by feedback regulation to compensate for the loss of important functional proteins. The alluvial diagram shows the dynamics of the differentially expressed transcripts from the PreL to LZ stages (Figure 7G). Specifically, most of the transcripts that were up-regulated from PreL to LZ in the WT were down-regulated in the LZ of *Nat10*-SKO cells (98.56%, *n* = 2017), and the transcripts that were down-regulated from PreL to LZ in the WT were up-regulated in *Nat10*-SKO LZ meiocytes (98.96%, *n* = 673). In addition, a subset of the transcripts stably expressed from PreL to LZ in the WT were down-regulated (56.17%, *n* = 3208) or up-regulated (43.83%, *n* = 3208) in the LZ of *Nat10*-SKO cells (Figure 7G). Moreover, the expression levels of some key genes involved in synapsis, meiotic recombination, DSB repair and piRNA pathway processes were significantly reduced in the LZ in the absence of NAT10 (Figure 7H and Supplementary Figure S9E). These results demonstrated that NAT10 deletion causes transcriptional dysregulation, especially the down-regulation of key genes involved in meiosis, which ultimately leads to male sterility.

### Loss of *Nat10* leads to reduced abundance of ac^4^C modification

**Figure 8.**
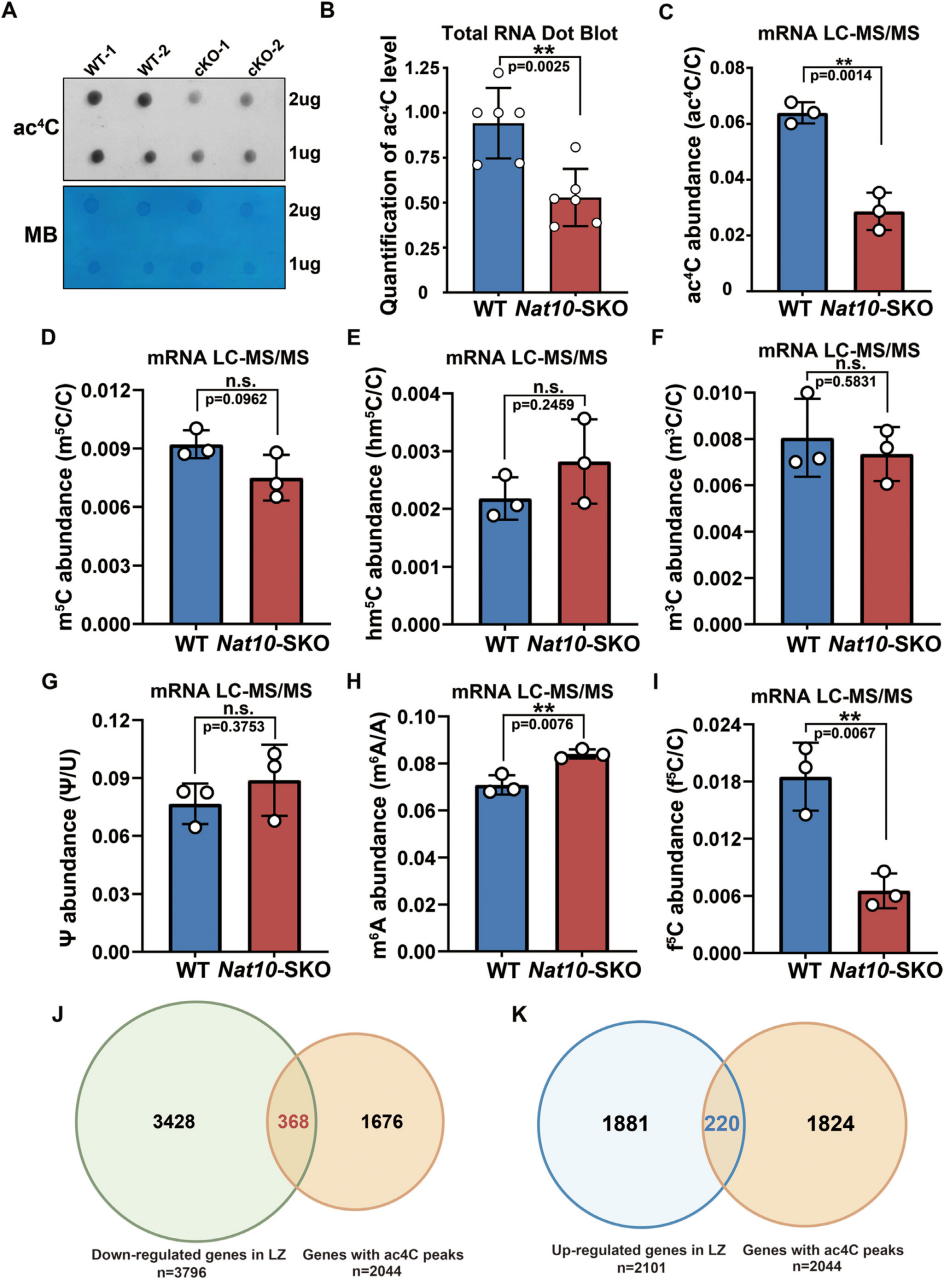
NAT10 depletion reduces ac^4^C abundance and results in dysregulation of functional genes in spermatogenesis. (**A**) Dot blotting of ac^4^C modification in WT and *Nat10-*SKO 12 dpp mice testes. Methylene blue staining was used as the total RNA loading control. (**B**) Quantification of ac^4^C modifications in total RNA from the control testes or *Nat10*-SKO testes relative to (A). The experiments were repeated three times. Mean ± SEM. **P < 0.01 via two-tailed Student's *t*-test. (**C**) ac^4^C detection in WT and *Nat10-*SKO testicular mRNA using LC-MS/MS. Mean ± SEM. The three biological replicates are represented by dots. Mean ± SEM. **P < 0.01. (**D–I**) LC-MS/MS detected the relative m^5^C/C, hm^5^C/C, m^3^C/C, ψ/U, m^6^A/A and f^5^C/C abundance in purified mRNA from WT and *Nat10-*SKO testes. Each dot represents a biological replicate. *n* = 3. Error bars indicate SEM. **P < 0.01 by two-tailed Student's *t*-test. n.s. means not significant. (**J**) Venn plot showing the overlap of down-regulated transcripts in *Nat10*-SKO leptotene/zygotene (L/Z) cells and genes with ac^4^C peaks, as previously published (23) (GSE102113). (**K**) Venn diagrams showing the overlap of up-regulated genes in *Nat10-*SKO leptotene/zygotene (L/Z) cells and genes with ac^4^C peaks, as previously published (GEO: GSE102113).

Next, we used dot blotting and HPLC-MS/MS to detect changes in ac^4^C in the testes of *Nat10*-SKO male mice. First, 12 dpp testicular total RNA was detected using dot blot analysis, and it was found that the abundance of ac^4^C was significantly reduced after NAT10 deletion (Figure 8A and B). Furthermore, 12 dpp testicular mRNA was detected using HPLC-MS/MS, wherein ac^4^C was also found to be significantly reduced but not completely abolished (Figure 8C and Supplementary Figure S10A–D). To determine whether ac^4^C modifications are affected by NAT10 gene disruption, we detected other RNA modifications using HPLC-MS/MS, and the results showed no significant difference in m^5^C, hm^5^C, m^3^C and Ψ modification levels in *Nat10*-SKO testis mRNA compared with controls (Figure 8D–G), thus indicating that decreased ac^4^C levels were regulated by NAT10 rather than by meiotic arrest. Surprisingly, we also found that m^6^A levels were significantly increased in *Nat10*-SKO testis mRNA (Figure 8H), whereas f^5^C levels were significantly diminished (Figure 8I). This finding suggests that there may be a cross-talk mechanism between different mRNA modifications. Previous studies have reported that 2044 transcripts contain ac^4^C peaks in mammals (23). We then compared the DEGs in the LZ with the ac^4^C-containing genes in WT HeLa cells and found that 368 genes were shared between the down-regulated genes in LZ (*n* = 3796) and ac^4^C-containing genes (*n* = 2044) (Figure 8J). GO analysis of overlapping genes (*n* = 368) revealed that most were enriched in DNA damage response, DNA repair, chromatin remodeling and the DNA recombination process (Supplementary Figure S10E and Supplementary Table S7). Similarly, 220 genes were shared between the up-regulated genes in LZ (*n* = 2101) and genes containing ac^4^C (*n* = 2044) (Figure 8K). GO enriched these 220 genes and found that most of them engaged in transcriptional regulation (Supplementary Figure S10F and Supplementary Table S7). These results indicated that the abnormal phenotype after NAT10 deletion may be due to an imbalance in the expression of genes containing ac^4^C peaks.

## DISCUSSION

In this study, we first detected the expression of NAT10, the only known ac^4^C writer protein, in different tissues and found that NAT10 was preferentially expressed in reproductive organs such as the testes, ovaries and epididymis (Figure 1). Furthermore, we used dot blotting and HPLC-MS/MS to detect ac^4^C modifications in different tissues. The results showed that the chemical content of ac^4^C was higher in the testes, ovaries and epididymis (Figure 2). This prompted us to further explore the dynamic changes and functions of ac^4^C modifications in germ cells. As a result, we constructed a germ cell-specific *Nat10* knockout mouse model to study the physiological functions of ac^4^C modifications *in vivo* (Figure 3). Conditional inactivation of *Nat10* reduced the level of ac^4^C stoichiometry, especially the reduction of ac^4^C modification in the functional genes of spermatogenesis (Figure 8). Transcriptome profiling revealed that the deletion of NAT10 led to many dysregulated transcripts (Figure 7). This seriously affected spermatogonial differentiation and meiotic entry (Figure 4 and Supplementary Figures S5 and S6), and led to defects in the assembly of synaptic complexes, homologous recombination and DSB repair (Figures 5 and 6), ultimately resulting in male sterility. Our study revealed dynamic changes in ac^4^C modifications during spermatogenesis and reported the physiological function of NAT10 in mammals *in vivo*.

The m^6^A RNA modification has been demonstrated by compelling evidence to be essential for mouse spermatogenesis. Studies on knockout models of m^6^A-associated factors, including writers, readers and erasers, have revealed that m^6^A participates in the precise regulation of spermatogonial differentiation, meiosis and spermiogenesis (55,56). Germ cell-specific ablation of the m^6^A methyltransferase complex core catalytic subunit protein METTL3 or scaffolding protein METTL14 with *Vasa-Cre* causes exhaustion of the spermatogonial stem cell pool due to excessive spermatogonial proliferation (37,57). However, the combined deletion of *Mettl3* and *Mettl14* using *Stra8-GFPCre* resulted in defective spermiogenesis (but normal meiosis) (37). Mechanistically, METTL3- and METTL14-mediated m^6^A modifications regulate the precise alternative splicing and timely translation of methylated transcripts functioning in spermatogenesis, which are essential for spermatogenesis and male fertility (37,57). Notably, ac^4^C writers have many similarities to and differences from m^6^A writers. Similarly, NAT10 and METTL3/METTL14 are localized in the nucleus of male germ cells. In addition, the establishment of ac^4^C and m^6^A is highly regulated, and their abundance changes dynamically during spermatogenesis. With germ cell-specific inactivation of *Nat10*, both *Mettl3* and *Mettl14* can cause defects in spermatogenesis and male infertility. This suggests that although the abundance of mRNA modification is relatively low compared with that of DNA modification and histone modification, it still plays an irreplaceable physiological role in male germ cell development. The differences were as follows: first, the expression level of NAT10 in each stage of spermatogenesis was significantly higher than that of m^6^A methyltransferase (METTL3, METTL14 and WTAP), and their expression patterns were inconsistent (Supplementary Figure S2C). Second, *Stra8-GFPCre*-mediated single inactivation of *Mettl3* or *Mettl14* does not affect meiosis, and both single conditional knockout mice were fertile and exhibited normal spermatogenesis. Interestingly, a single conditional *Nat10* knockout can cause more serious defects in spermatogonial differentiation, meiotic entry and meiosis than a double conditional knockout of *Mettl3* and *Mettl14* with the same *Stra8-GFPCre*. Although deletion of both *Mettl3* and *Mettl14* significantly reduced the level of m^6^A in meiosis-associated transcripts, it did not affect the meiotic process. This may be due to the m^6^A methyltransferase complex containing multiple components, and the presence of functional redundancy among them. In our study, the inactivation of *Nat10* resulted in a decrease in the abundance of ac^4^C in key genes involved in spermatogenesis and caused severe meiotic defects. Furthermore, ac^4^C was not completely eliminated after the deletion of *Nat10*. This suggests that the function of ac^4^C modification may be more essential than that of m^6^A modification during meiosis.

Nevertheless, we did not have direct evidence to clarify how the reduction in ac^4^C modification after *Nat10* deletion leads to the dysregulation of many transcripts. We hypothesize that the following mechanisms may exist. (i) NAT10 is the only known ac^4^C writer so far, and it is well established in cultured cell lines that *Nat10* depletion results in decreased ac^4^C levels and that ac^4^C modification could enhance mRNA stability (23,24). In our study, ac^4^C levels decreased during spermatogenesis in *Nat10*-SKO mice, resulting in reduced mRNA stability and eventual degradation. This is one possible mechanism of transcript down-regulation. (ii) Regarding the mechanism of gene up-regulation after *Nat10* deletion, we suggest that mRNAs chemically modified by ac^4^C may be recognized by different readers, thus mediating mRNA splicing and processing, transport out of the nucleus and translational activation and degradation (58–60). When the ac^4^C modification is reduced, readers cannot efficiently recognize and bind RNA, which may lead to abnormal splicing, defective transport and translation, and failure to decay in good time (5,25,61). (iii) Another possible mechanism is that ac^4^C modification occurs on mRNAs encoding transcription factors. When ac^4^C modifications are reduced, these mRNAs are dysregulated, thereby indirectly affecting global transcriptional activity.

High-throughput sequencing techniques (acRIP-Seq or ac^4^C-seq) can be used to map the dynamic patterns of ac^4^C during spermatogenesis and identify changes in ac^4^C (peak distribution and corresponding genes) after *Nat10* deletion, which will better address these scientific questions and help to understand the underlying mechanism. However, the current acRIP-Seq (62) and ac^4^C-Seq (63) technologies require a high initial amount of RNA. The number of spermatocytes in *Nat10*-SKO mice is small, which is an unprecedented challenge for sorting specific stages of cells with high purity and adequate quantity to carry out acRIP-Seq or ac^4^C-Seq. However, acRIP-Seq technology cannot achieve single-nucleotide resolution profiling. Therefore, ac^4^C mapping technologies with high-sensitivity, single-nucleotide resolution and low sample quantity requirements need to be developed to study the distribution and regulatory mechanism of ac^4^C modifications in rare samples such as germ cells, embryos and clinical disease samples.

## DATA AVAILABILITY

Raw RNA-seq data were deposited in the NCBI Gene Expression Omnibus database (https://www.ncbi.nlm.nih.gov/geo/query/acc.cgi?acc = GSE191105). The GEO accession number GSE191105 with the password ‘mnsfuyowplavtev’ was used.

## Supplementary Material

gkac594_Supplemental_Files
