# Supplementary material for: NAT10-mediated N4-acetylcytidine modification is required for meiosis entry and progression in male germ cells
Source: Nucleic Acids Res. 2022 Jul 8;50(19):10896–913. doi: 10.1093/nar/gkac594 (PMC9638909; doi:10.1093/nar/gkac594)

Figure 1 A

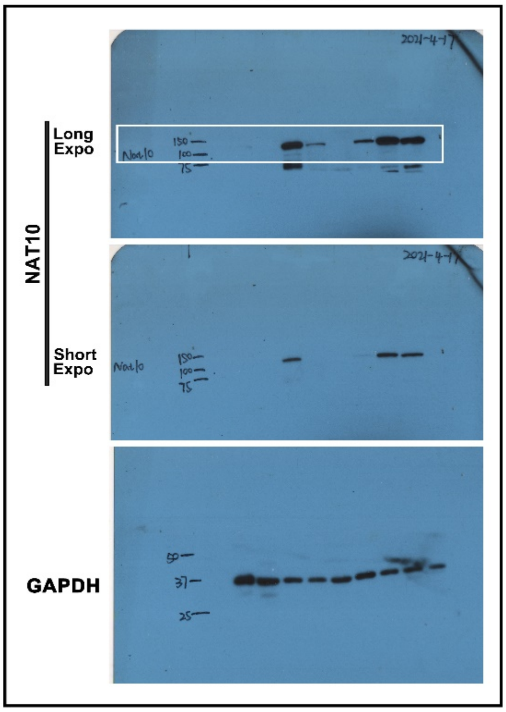

Figure 1 D

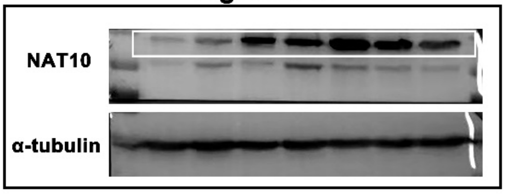

Figure 1 E

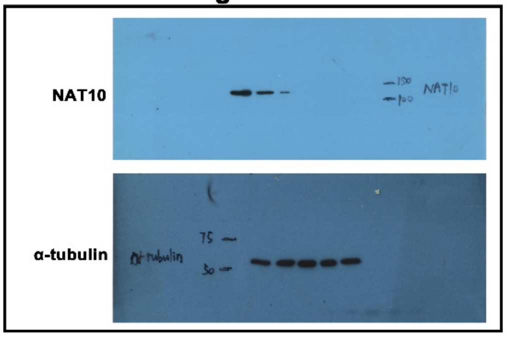

Figure 1 C

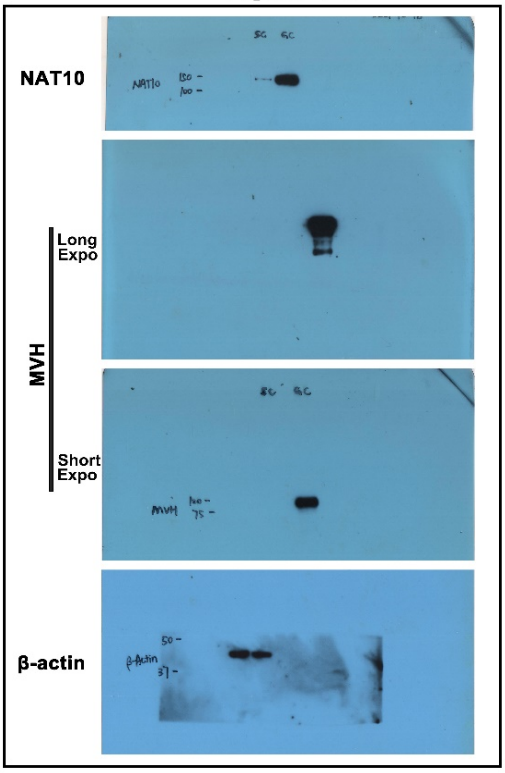

Figure 1 D

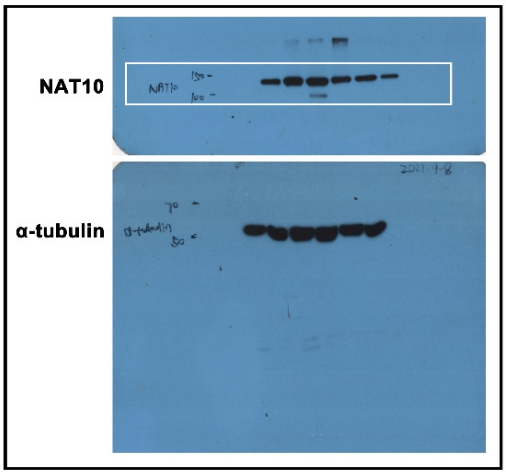

Figure 2 A

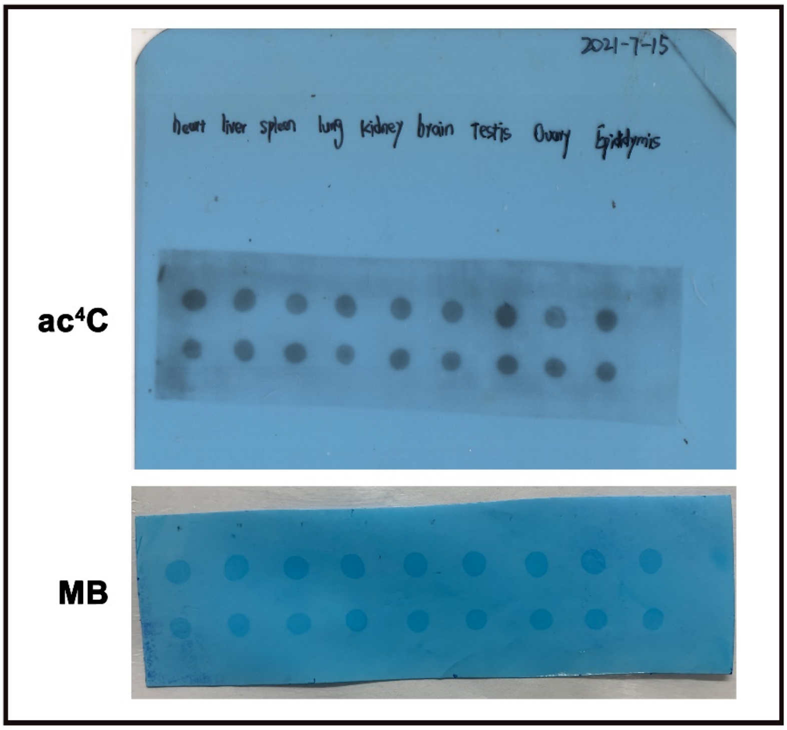

Figure 3 C

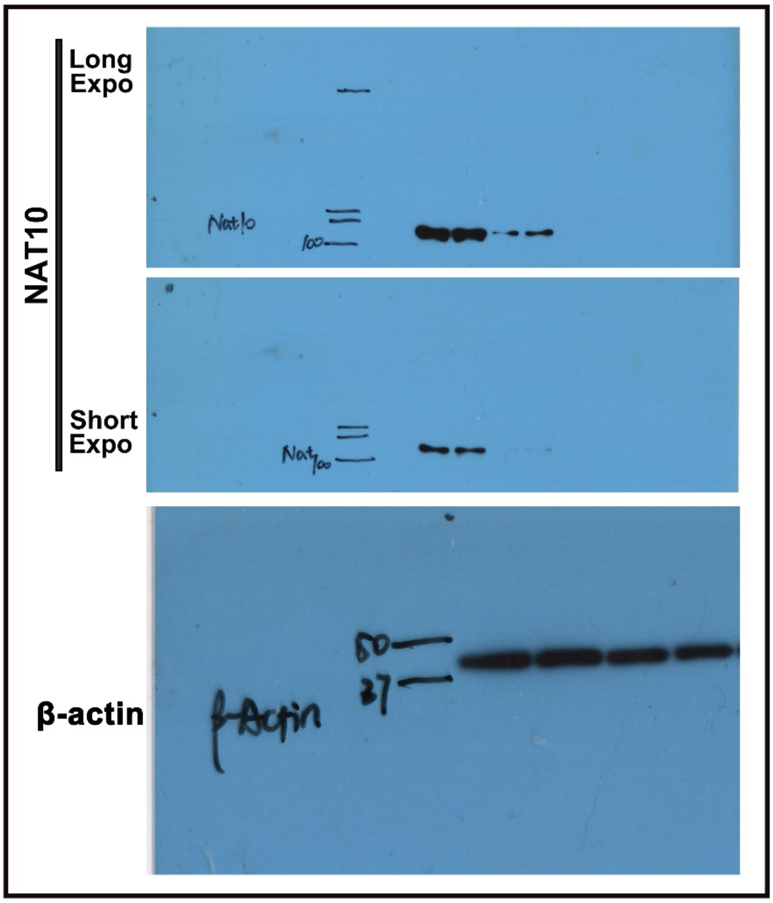

Figure 4 H

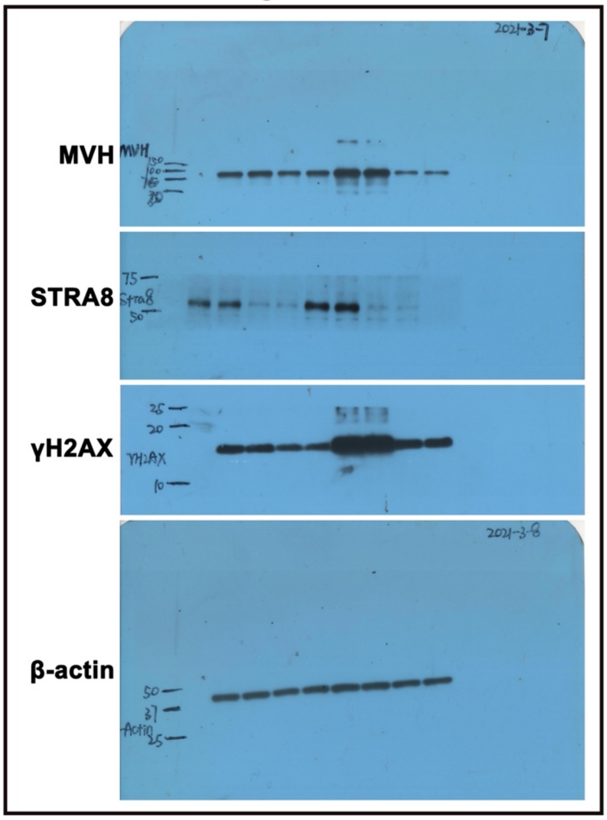

Figure 4 I

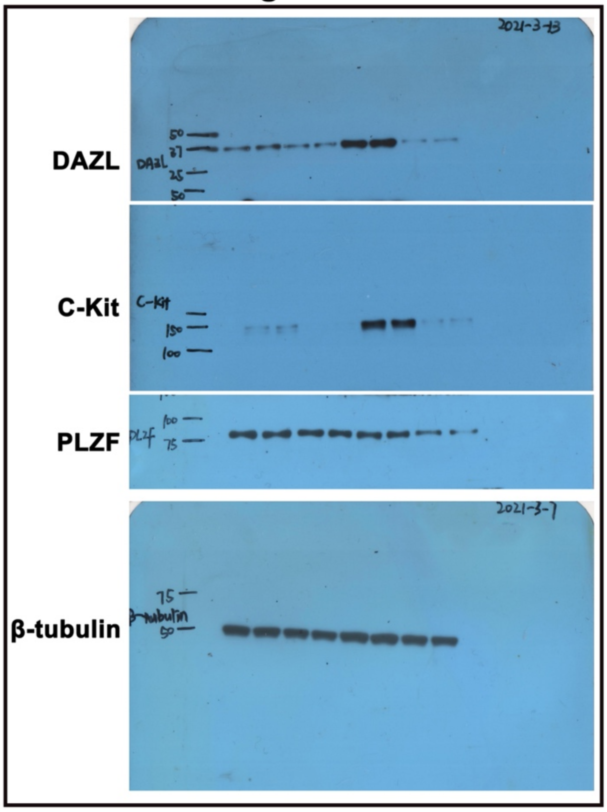

Figure 4 J

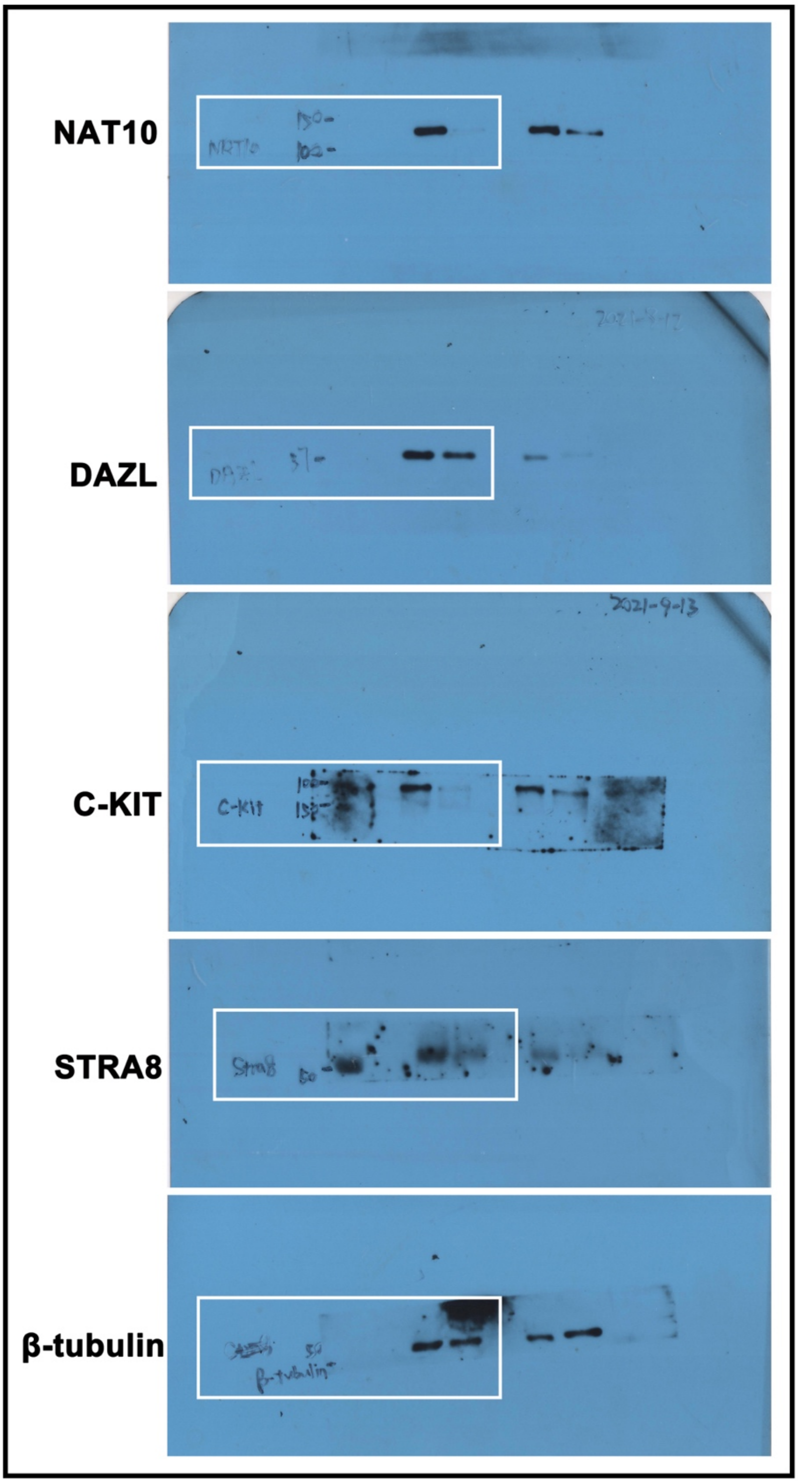

Figure 7A

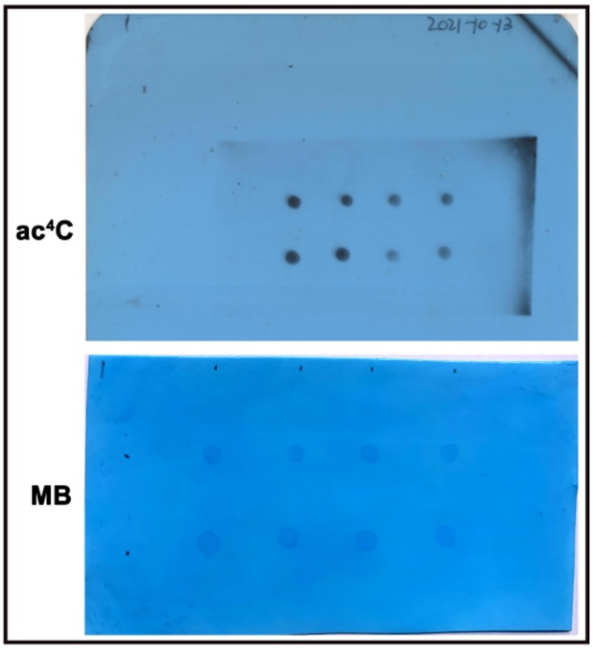

Figure S 7 F

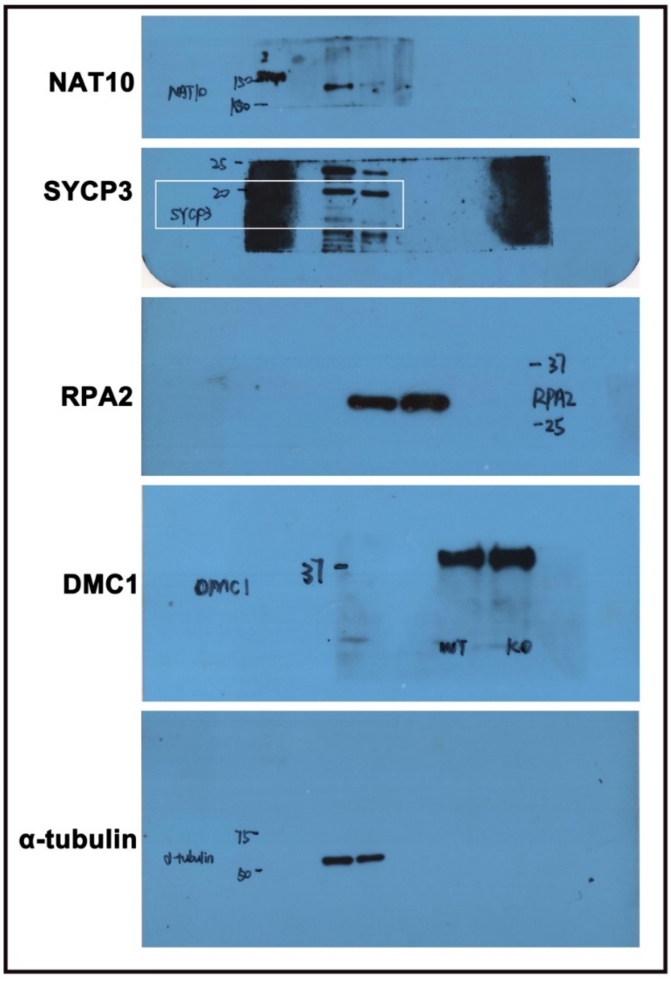

Supplement: gkac594_Supplemental_Files [file gkac594_supplemental_files.zip › Supplementary Figure S11. Unprocessed gel figures.pdf]
