# Supplementary material for: NAT10-mediated N4-acetylcytidine modification is required for meiosis entry and progression in male germ cells
Source: Nucleic Acids Res. 2022 Jul 8;50(19):10896–913. doi: 10.1093/nar/gkac594 (PMC9638909; doi:10.1093/nar/gkac594)
Supplement: gkac594_Supplemental_Files [file gkac594_supplemental_files.zip › Supplementary Materialsú¿Supplementary Figure S1-10 and Supplementary Tables S1-S2ú⌐.pdf]

Supplementary Materials

NAT10-mediated N^4-Acetylcytidine Modification is Required for Meiosis Entry and Progression in Male Germ Cells

Lu Chen[1,†], Wen-Jing Wang[1,†], Qiang Liu[2,†], Yu-Ke Wu[1,†], Yun-Wen Wu[1], Yu Jiang[1], Xiu-Quan Liao[3], Fei Huang[1], Yang Li[1], Li Shen[1], Chao Yu[4,5], Song-Ying Zhang[4], Li-Ying Yan[2], Jie Qiao[2,*], Qian-Qian Sha[3,*], Heng-Yu Fan[1,4,*]

This combined PDF file contains the following figures and tables:

Figure S1. NAT10 is highly conserved in different species and has a conserved N-acetyltransferase domain
Figure S2. Expression of NAT10 during testicular development and spermatogenesis in mice
Figure S3. The dynamic localization of NAT10 in different stages of spermatocytes
Figure S4. *Nat10* conditional knockout strategy and *Nat10*-SKO mouse characterization
Figure S5. NAT10 is crucial for the meiosis entry
Figure S6. NAT10 is crucial for spermatogonial differentiation
Figure S7. NAT10 is essential for meiotic prophase I
Figure S8. Purity quantification after cell sorting
Figure S9. Loss of NAT10 causes transcriptional dysregulation
Figure S10. *Nat10* deficiency reduces ac^4C modification abundance
Supplementary Table S1. Antibodies used in this study.
Supplementary Table S2. List of primer sequences related to experimental procedures

The following supplementary files have been uploaded separately：

Figure S11. Unprocessed gel figures
Table S3. ERCC-calibrated counts and TPM list
Table S4. Correlation of all samples
Table S5. List of differentially expressed genes in WT and *Nat10*-SKO
Table S6. List of GO enrichment of differentially expressed genes in WT and *Nat10*-SKO
Table S7. GO List of overlap genes between LZ stage DEGs with ac^4C-transcripts in HeLa cells

Supplementary Figures

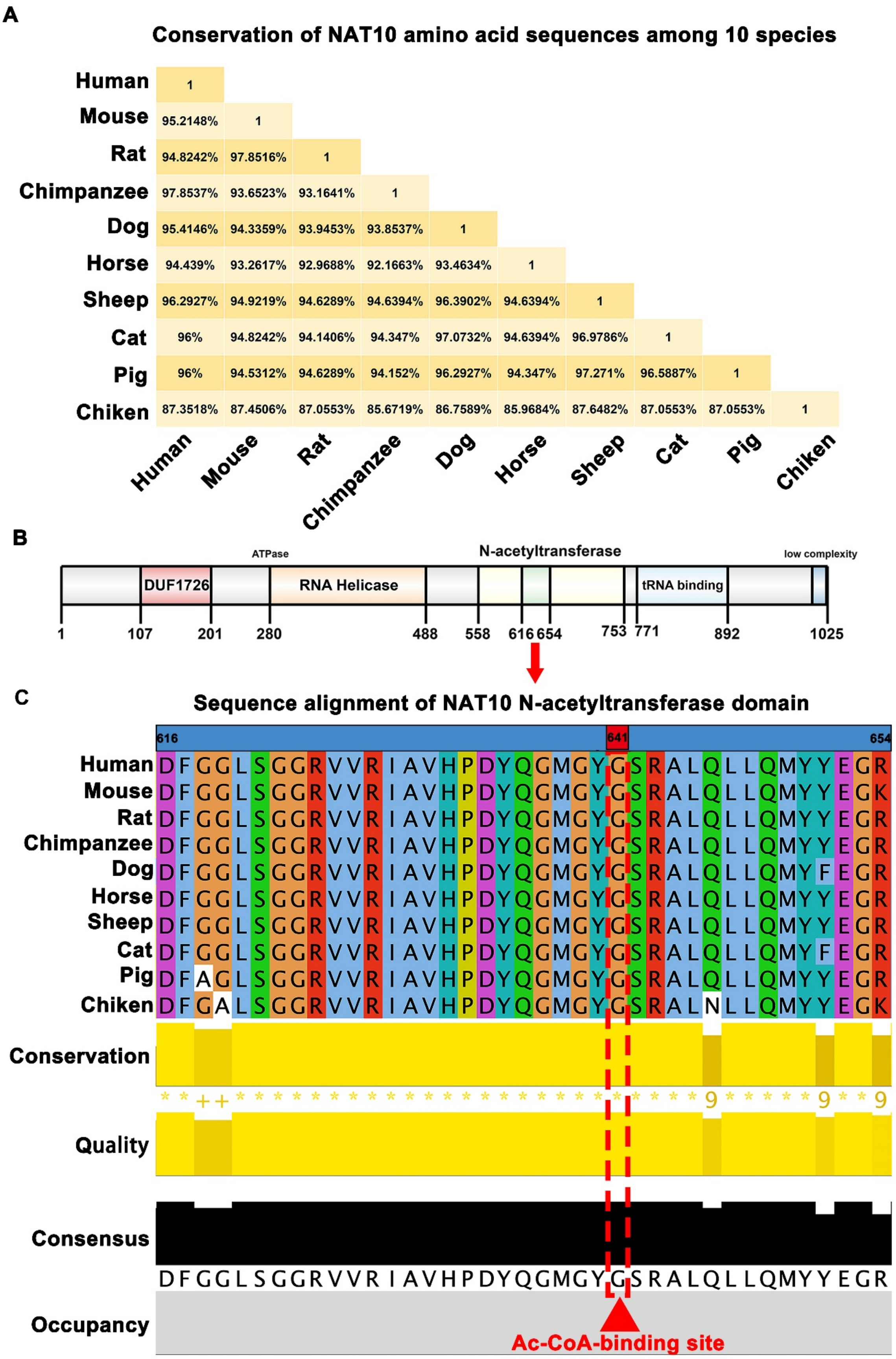

Figure S1. NAT10 is highly conserved in different species and has a conserved N-acetyltransferase domain

A: Sequence alignment of NAT10 amino acids among 10 species.

B: Representation of NAT10 with its known domains.

C: N-acetyltransferase domain alignment of NAT10, showing highly conserved sequences among species. G641 was identified as an ac-CoA-binding site.

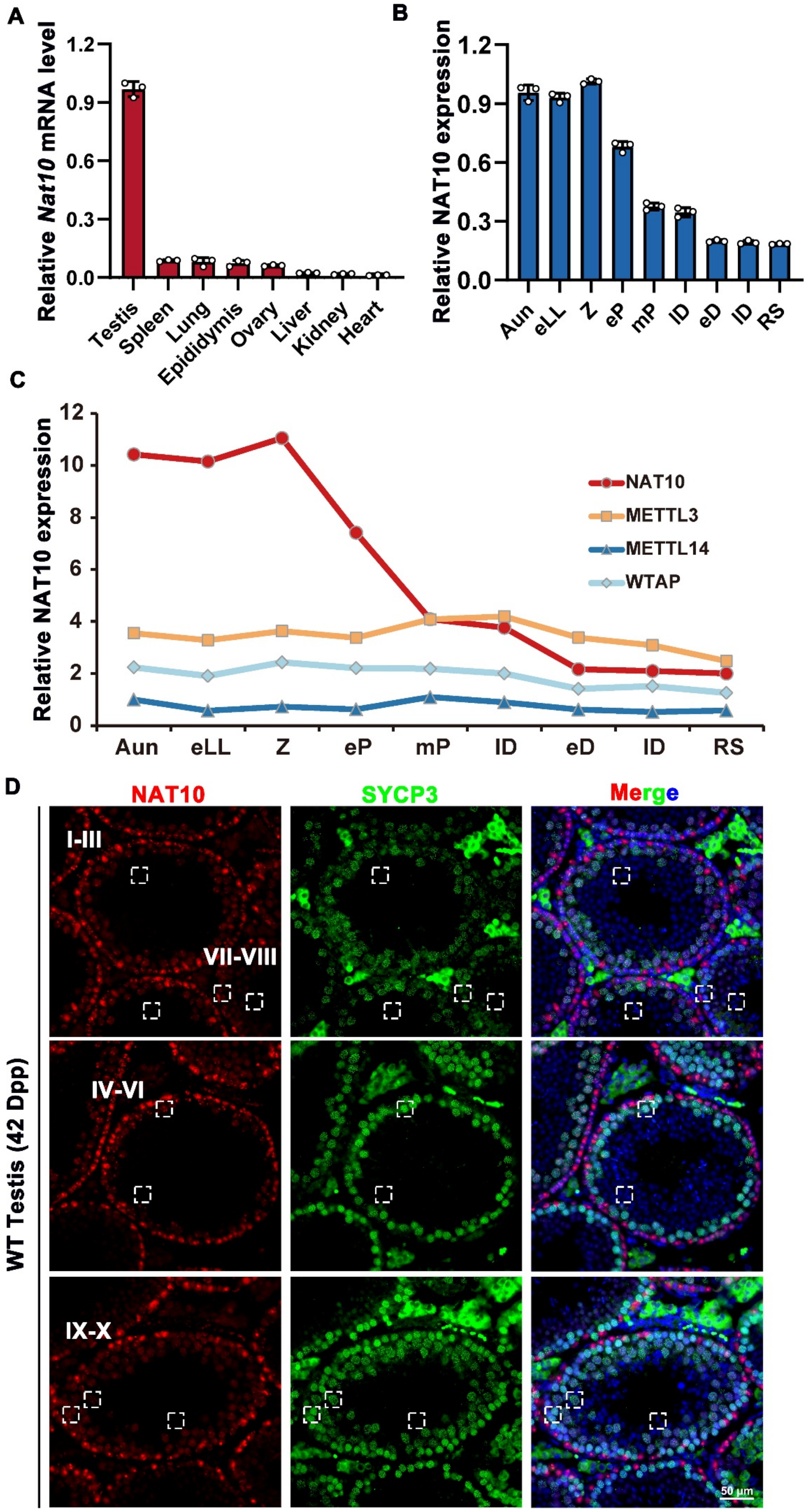
A
Relative Nat10 mRNA level
1.2
0.9
0.6
0.3
0.0
Testis
Spleen
Lung
Epididymis
Ovary
Liver
Kidney
Heart
B
Relative NAT10 expression
1.2
0.9
0.6
0.3
0.0
Aun
eLL
Z
eP
mP
ID
eD
ID
RS
C
Relative NAT10 expression
12
10
8
6
4
2
0
NAT10
METTL3
METTL14
WTAP
Aun
eLL
Z
eP
mP
ID
eD
ID
RS
D
NAT10
SYCP3
Merge
I-III
VII-VIII
IV-VI
IX-X
WT Testis (42 Dpp)
50 μm

Figure S2. Expression of NAT10 during testicular development and spermatogenesis in mice

A: Quantitative reverse-transcription PCR (RT-qPCR) analysis of *Nat10* mRNA levels in various organs of adult mice. Data are presented as an expression relative to that of the testis and normalized to *GAPDH* expression in each organ. Error bars indicate mean ± SEM.

B: Relative NAT10 protein expression levels during spermatogenesis. Data were extracted from published proteomics results.

C: Relative protein expression levels of NAT10 and m^6A writers during mouse spermatogenesis. Data were extracted from published proteome results.

D: Co-immunostaining of NAT10 and SYCP3 in WT 42 dpp mouse testis related to the data in Figure 1F. Scale bar = 50 μm.

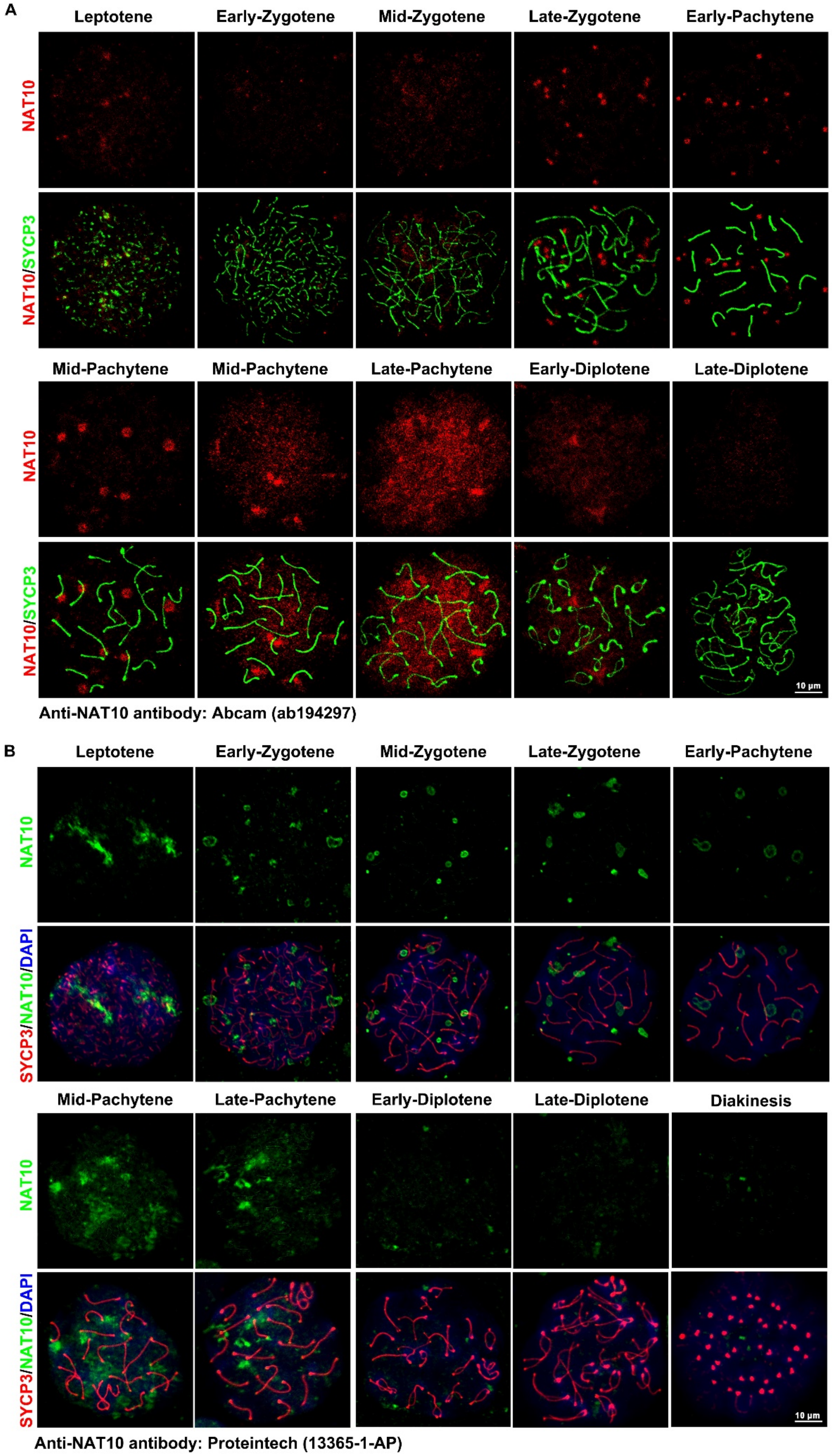
A
Leptotene
Early-Zygotene
Mid-Zygotene
Late-Zygotene
Early-Pachytene
NAT10
NAT10/SYCP3
Mid-Pachytene
Mid-Pachytene
Late-Pachytene
Early-Diplotene
Late-Diplotene
NAT10
NAT10/SYCP3
10 μm
Anti-NAT10 antibody: Abcam (ab194297)
B
Leptotene
Early-Zygotene
Mid-Zygotene
Late-Zygotene
Early-Pachytene
NAT10
SYCP3/NAT10/DAPI
Mid-Pachytene
Late-Pachytene
Early-Diplotene
Late-Diplotene
Diakinesis
NAT10
SYCP3/NAT10/DAPI
10 μm
Anti-NAT10 antibody: Proteintech (13365-1-AP)

Figure S3. The dynamic localization of NAT10 in different stages of spermatocytes

A and **B:** Immunostaining of nuclear spreading with two kinds of anti-NAT10 antibodies (A: Abcam, ab194297; B: Proteintech, 13365-1-AP). The meiotic stages of spermatocytes were determined by SYCP3 staining of the chromosomal axes. Scale bar = 10 μm.

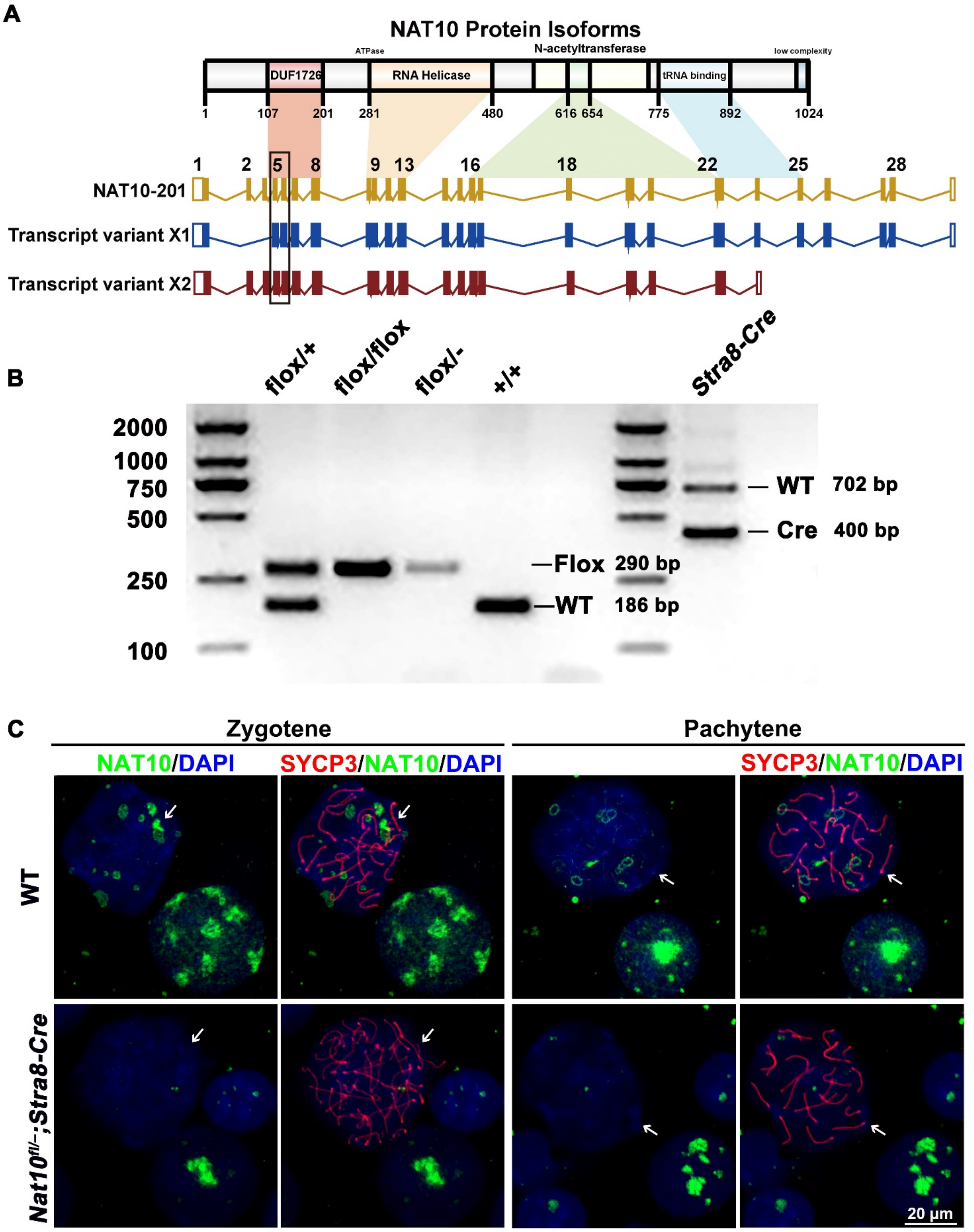
A
NAT10 Protein Isoforms
ATPase
N-acetyltransferase
low complexity
DUF1726
RNA Helicase
tRNA binding
1
107
201
281
480
616 654
775
892
1024
1 2 5 8 9 13 16 18 22 25 28
NAT10-201
Transcript variant X1
Transcript variant X2
B
flox/+
flox/flox
flox/-
+/+
Stra8-Cre
2000
1000
750
500
250
100
WT 702 bp
Cre 400 bp
Flox 290 bp
WT 186 bp
C
Zygotene
Pachytene
NAT10/DAPI
SYCP3/NAT10/DAPI
WT
Nat10fl/−;Stra8-Cre
20 µm

Figure S4. *Nat10* conditional knockout strategy and *Nat10*-SKO mouse characterization

A: Schematic diagram of three transcript variants of the mouse *Nat10* gene, and these three variants also share both the fourth and fifth exon.

B: Genotype identification of *Nat10* conditional knockout mice using the *Nat10-Flox* and *Stra8-Cre* primers. The primer sequences are listed in Supplementary Table S2.

C: Knockout efficiency verification via immunofluorescence staining using an anti-NAT10 antibody. SYCP3 was co-stained to indicate the spermatocytes. Scale bar = 10 μm.

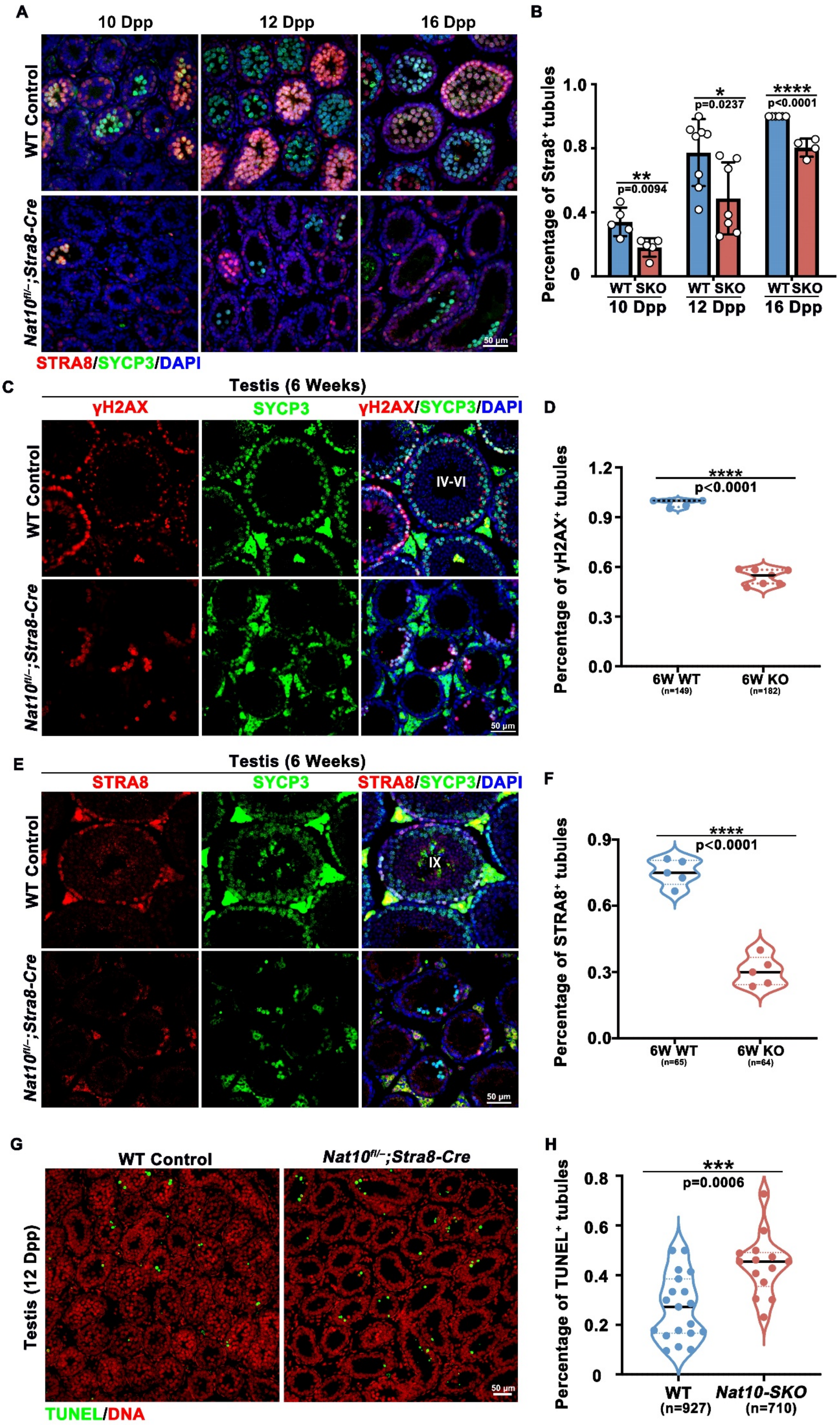
A
10 Dpp
12 Dpp
16 Dpp
WT Control
Nat10fl/−;Stra8-Cre
50 μm
STRA8/SYCP3/DAPI
B
Percentage of Stra8+ tubules
**
p=0.0094
*
p=0.0237
****
p<0.0001
WT SKO
10 Dpp
12 Dpp
16 Dpp
C
Testis (6 Weeks)
γH2AX
SYCP3
γH2AX/SYCP3/DAPI
IV-VI
D
Percentage of γH2AX+ tubules
****
p<0.0001
6W WT (n=149)
6W KO (n=182)
E
Testis (6 Weeks)
STRA8
SYCP3
STRA8/SYCP3/DAPI
IX
F
Percentage of STRA8+ tubules
****
p<0.0001
6W WT (n=65)
6W KO (n=64)
G
WT Control
Nat10fl/−;Stra8-Cre
Testis (12 Dpp)
TUNEL/DNA
H
Percentage of TUNEL+ tubules
***
p=0.0006
WT (n=927)
Nat10-SKO (n=710)

Figure S5. NAT10 is crucial for the meiosis entry

A: Immunofluorescence staining of STRA8 (red) and SYCP3 (green) in WT control and *Nat10*-SKO testes at the different stages indicated above. Scale bar = 50 μm.

B: Statistics of the ratio of STRA8-positive ($STRA8^+$) tubules in control and *Nat10*-SKO testes related to (A). Data are presented as mean ± SEM. * indicates $p < 0.1$, ** indicates $p < 0.01$, **** indicates $p < 0.0001$

C-D: Immunofluorescence co-staining (C) for γH2AX (red) and SYCP3 (green) in control and *Nat10*-SKO testes at 6weeks and quantification (D) of the ratio of γH2AX-positive ($\gamma H2AX^+$) tubules.

E-F: Immunofluorescence staining (E) of STRA8 (red) and SYCP3 (green) and quantification (F) of the ratio of STRA8-positive ($STRA8^+$) tubules in 6 weeks control and *Nat10*-SKO testes. Data are presented as mean ± SEM. **** indicates $p < 0.0001$

G and **H:** Sections from WT and *Nat10*-SKO testes were stained with TUNEL kit (green) to determine the number of apoptotic cells, scale bar = 50 μm (G). Quantification of apoptotic tubules (TUNEL-positive cells). Data are presented as mean ± SEM. *** indicates $p < 0.001$ (H).

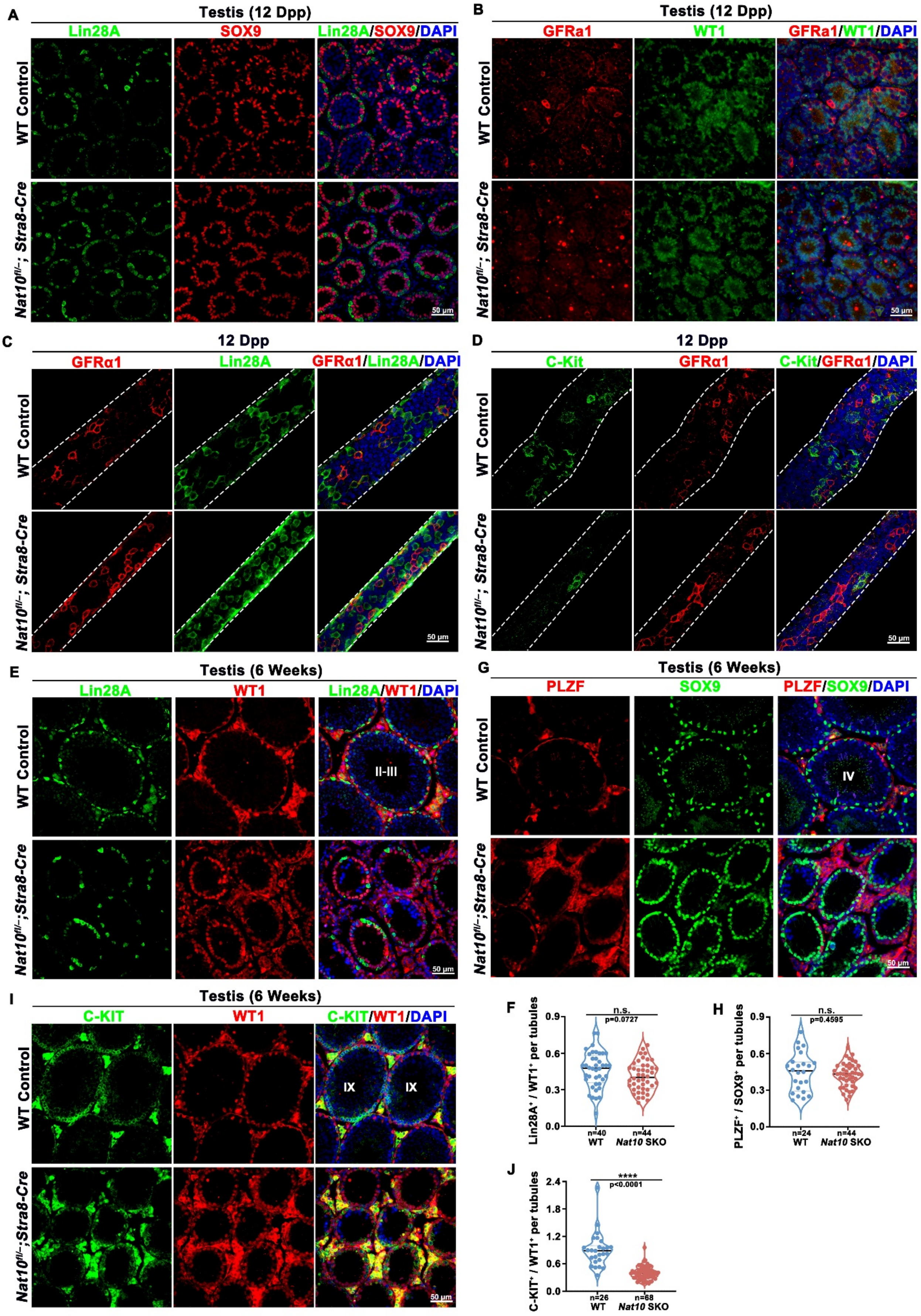

A
Testis (12 Dpp)
Lin28A
SOX9
Lin28A/SOX9/DAPI
WT Control
Nat10fl/-; Stra8-Cre
50 μm
B
Testis (12 Dpp)
GFRa1
WT1
GFRa1/WT1/DAPI
WT Control
Nat10fl/-; Stra8-Cre
50 μm
C
12 Dpp
GFRα1
Lin28A
GFRα1/Lin28A/DAPI
WT Control
Nat10fl/-; Stra8-Cre
50 μm
D
12 Dpp
C-Kit
GFRα1
C-Kit/GFRα1/DAPI
WT Control
Nat10fl/-; Stra8-Cre
50 μm
E
Testis (6 Weeks)
Lin28A
WT1
Lin28A/WT1/DAPI
WT Control
II-III
Nat10fl/-;Stra8-Cre
50 μm
G
Testis (6 Weeks)
PLZF
SOX9
PLZF/SOX9/DAPI
WT Control
IV
Nat10fl/-;Stra8-Cre
50 μm
I
Testis (6 Weeks)
C-KIT
WT1
C-KIT/WT1/DAPI
WT Control
IX
IX
Nat10fl/-;Stra8-Cre
50 μm
F
n.s.
p=0.0727
Lin28A+ / WT1+ per tubules
0.9
0.6
0.3
0.0
n=40
WT
n=44
Nat10 SKO
H
n.s.
p=0.4595
PLZF+ / SOX9+ per tubules
0.9
0.6
0.3
0.0
n=24
WT
n=44
Nat10 SKO
J
****
p<0.0001
C-KIT+ / WT1+ per tubules
2.4
1.8
1.2
0.6
0.0
n=26
WT
n=68
Nat10 SKO

Figure S6. NAT10 is crucial for spermatogonial differentiation

A: Immunostaining of LIN28A and SOX9 in histological sections of control and *Nat10*-SKO 12 dpp testes.

B: Immunostaining of seminiferous tubules for GFRα1 (red), a marker for early stages of undifferentiated spermatogonia, and Sertoli cell marker WT1 (green).

C and **D:** Whole-mount immunostaining of seminiferous tubules for GFRα1, LIN28A, and C-KIT in control and *Nat10*-SKO 12 dpp testes.

E-F: Immunostaining (E) and quantification (F) of Lin28A$^+$ cells/WT1$^+$ cells per tubule in sections from adult control and *Nat10*-SKO testes. n.s. indicates not significant via two-tailed Student's *t*-test.

G-H: Immunostaining (G) and quantification (H) of undifferentiated spermatogonia marker PLZF$^+$ cells/SOX9$^+$ cells per tubule in histological sections of control and *Nat10*-SKO 6 weeks testes. n.s. indicates not significant via two-tailed Student's *t*-test.

I-J: Immunostaining (I) and quantification (J) of the ratio of C-KIT$^+$ cells/WT1$^+$ cells per tubule in sections from 6 weeks control and germ-cell *Nat10*-mutant mice. **** indicates $p < 0.0001$ via two-tailed Student's *t*-test.

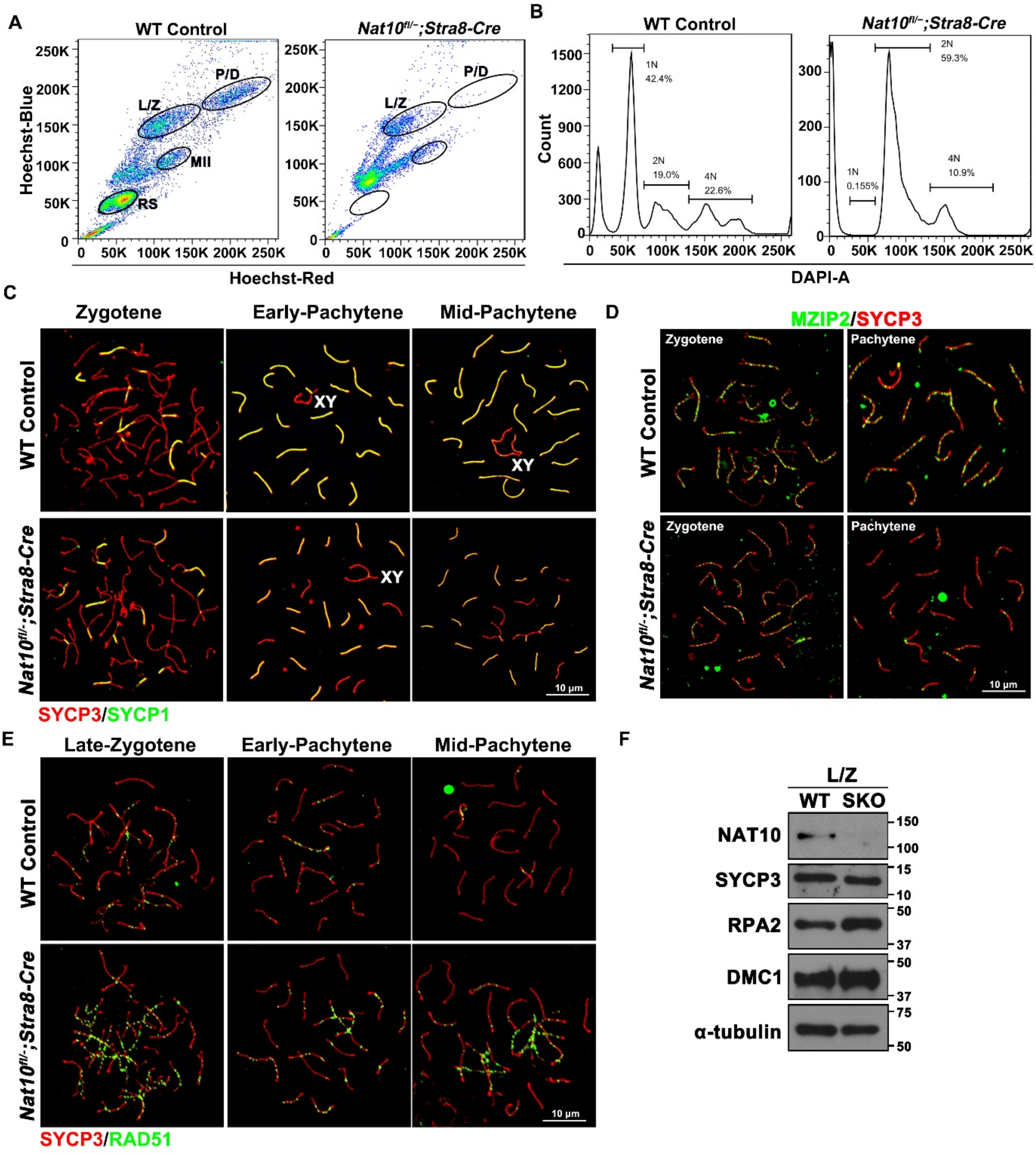

Figure S7. NAT10 is essential for meiotic prophase I

A: Representative fluorescence-activated cell sorting (FACS) profiles of individual spermatogenic populations based on Hoechst fluorescence from adult WT and *Nat10*-SKO male mice.

B: Representative FACS profiles from a 35-day-old mouse testicular cell suspension stained with Hoechst. Histogram peaks correspond to the N, 2N, and 4N cell populations.

C: Co-immunostaining of SYCP3 and SYCP1 in WT and *Nat10*-SKO mouse testes.

D: MZIP2 and SYCP3 were detected in the nuclear surface spreads of zygotene and pachytene spermatocytes.

E: Chromosome spreads of spermatocytes (late zygotene, early pachytene, and mid pachytene) from the testes of WT and *Nat10*-SKO males were immunostained for RAD51 and SYCP3.

F: Western blot detection of key protein levels in isolated leptotene/zygotene (L/Z) cells.

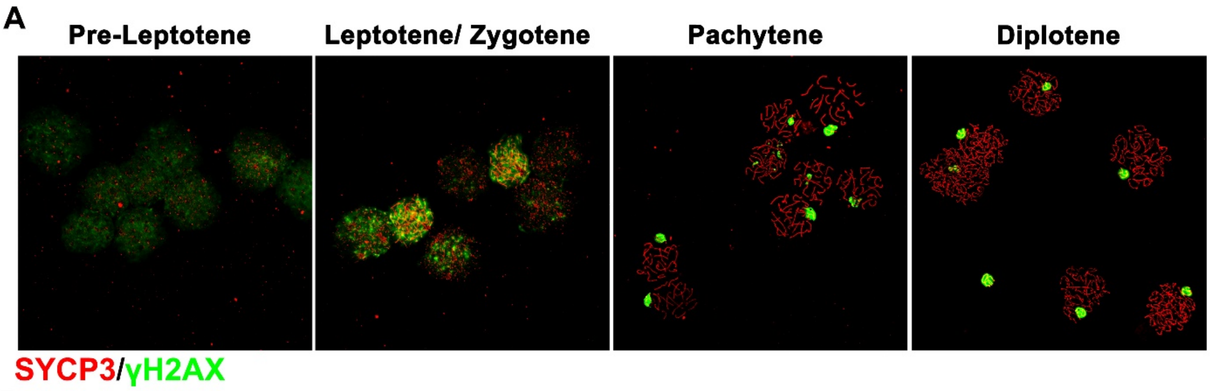

B

Collected \ Observed	SPG	Pre- Leptotene	Leptotene/ Zygotene	Pachytene	Diplotene	Others
Pre- Leptotene (n=1386)	35/1386 2.52%	1328/1386 95.82%	6/1386 0.43%	0	0	17/1386 1.23%
Leptotene/ Zygotene (n=1864)	4/1864 0.21%	144/1864 7.73%	1568/1864 84.12%	15/1864 0.80%	0	133/1864 7.14%
Pachytene (n=1660)	5/1660 0.30%	18/1660 1.08%	170/1660 10.24%	1387/1660 83.55%	20/1660 1.20%	60/1660 3.61%
Diplotene (n=1920)	0	4/1920 0.20%	87/1920 4.53%	143/1920 7.44%	1643/1920 85.57%	43/1920 2.24%

Figure S8. Purity quantification after cell sorting

A: Representative images showing sorted leptotene/zygotene (L/Z), pachytene (P), and diplotene (D) spermatocytes. The spread nuclei were double-labeled with γH2AX (green) and SYCP3 (red) and co-stained with DAPI (blue).

B: Percent purity quantification based on immunofluorescence analysis after cell sorting. Cell purity was calculated as (cell type observed/total cells) × 100%.

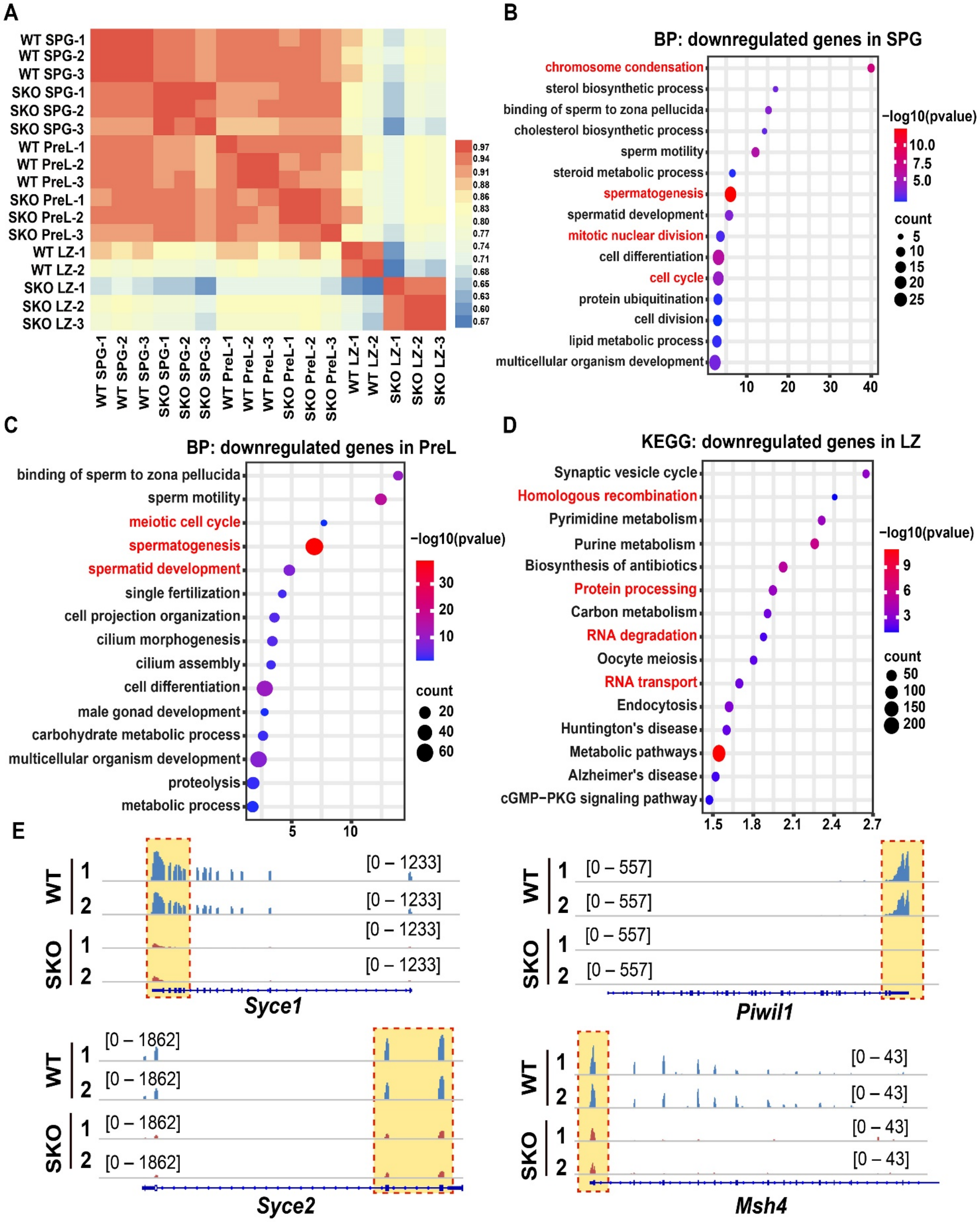
A
B
BP: downregulated genes in SPG
C
BP: downregulated genes in PreL
D
KEGG: downregulated genes in LZ
E
Syce1
Piwil1
Syce2
Msh4

Figure S9. Loss of NAT10 causes transcriptional dysregulation

A: Heatmap showing Pearson correlation coefficients of the total transcripts between WT and *Nat10*-SKO cells at the spermatogonia (SPG), pre-leptotene (PreL), and leptotene/zygotene (L/Z) stages, indicating a positive correlation between biological replicates. The color key indicates the value of the Pearson correlation coefficient from low (blue) to high (red).

B and **C:** Bubble plot showing the GO enrichment analysis of the identified downregulated genes in spermatogonia (SPG) (B) and pre-leptotene (PreL) (C) cells isolated from *Nat10*-SKO and WT mice in terms of biological processes.

D: KEGG pathway-based enrichment analysis of downregulated genes in leptotene/zygotene cells derived from *Nat10*-SKO and WT mice.

E: Genome browser snapshots showing the distribution of RNA-seq reads of selected representative genes. The differentially expressed exons of each gene are marked in yellow and shaded, and the intron/exon (box) genomic structure of each gene is shown in blue.

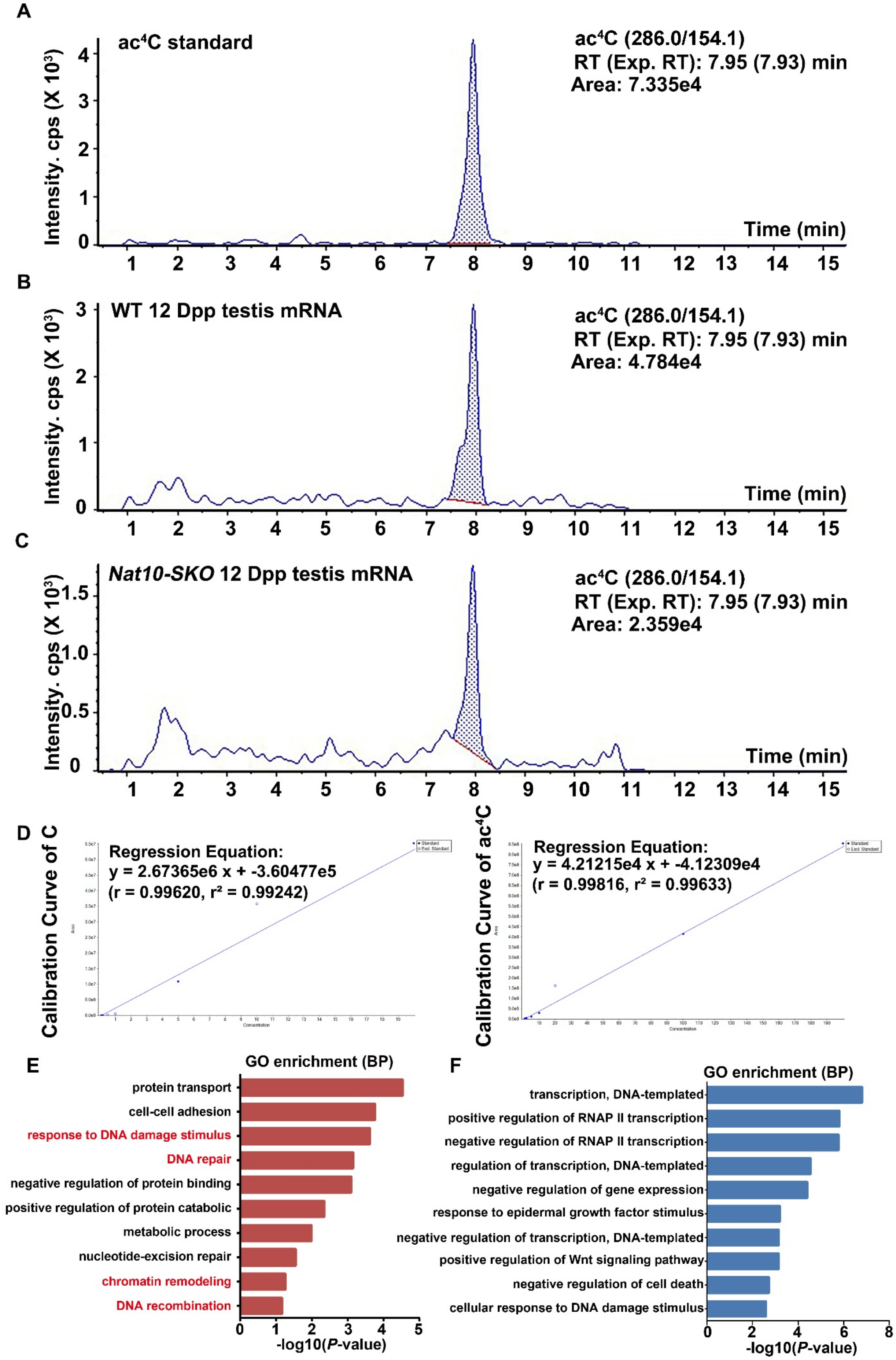
A
ac4C standard
ac4C (286.0/154.1)
RT (Exp. RT): 7.95 (7.93) min
Area: 7.335e4
Intensity. cps (X 10³)
Time (min)
B
WT 12 Dpp testis mRNA
ac4C (286.0/154.1)
RT (Exp. RT): 7.95 (7.93) min
Area: 4.784e4
C
Nat10-SKO 12 Dpp testis mRNA
ac4C (286.0/154.1)
RT (Exp. RT): 7.95 (7.93) min
Area: 2.359e4
D
Calibration Curve of C
Regression Equation:
y = 2.67365e6 x + -3.60477e5
(r = 0.99620, r² = 0.99242)
Calibration Curve of ac4C
Regression Equation:
y = 4.21215e4 x + -4.12309e4
(r = 0.99816, r² = 0.99633)
E
GO enrichment (BP)
protein transport
cell-cell adhesion
response to DNA damage stimulus
DNA repair
negative regulation of protein binding
positive regulation of protein catabolic
metabolic process
nucleotide-excision repair
chromatin remodeling
DNA recombination
-log10(P-value)
F
GO enrichment (BP)
transcription, DNA-templated
positive regulation of RNAP II transcription
negative regulation of RNAP II transcription
regulation of transcription, DNA-templated
negative regulation of gene expression
response to epidermal growth factor stimulus
negative regulation of transcription, DNA-templated
positive regulation of Wnt signaling pathway
negative regulation of cell death
cellular response to DNA damage stimulus
-log10(P-value)

Figure S10. ***Nat10*** **deficiency reduces ac^4C modification abundance**

A–C: LC-MS/MS chromatograms for the quantitation of ac^4C in ac^4C standards (A), WT testis mRNA (B), and *Nat10*-SKO testis mRNA (C).

D: Calibration curves used to quantitate cytidine and modified nucleosides by LC-MS/MS analysis.

E: Gene ontology (GO) analysis of the biological process category in the 368 overlapping genes in (Figure 8J).

F: GO analysis of the biological process category of the 220 overlapping genes in (Figure 8K).

Supplementary Table S1. Antibodies used in this study.

Antibody	Manufacture (catalog number)	Source	Applications (working dilution)
**SYCP3**	Abcam (ab97672)	Mouse	IF (1:200)<br>WB (1:1000)
**SYCP1**	Abcam (ab15087)	Rabbit	IF (1:200)<br>WB (1:1000)
**HORMAD1**	Proteintech (13917-1-AP)	Rabbit	IF: 1:300
**PLZF**	Santa Cruz (sc28319)	Rabbit	IF (1:200)<br>WB (1:1000)
**GFRα1**	R&D system (AF560)	Goat	IF (1:50)
**LIN28A**	R&D system (#AF3757)	Goat	IF (1:200)
**C-KIT**	Cell Signaling (D13A2)	Rabbit	IF (1:200)<br>WB (1:1000)
**SOX9**	Millipore (AB5535)	Rabbit	IF (1:300)
**WT1**	Abcam (ab212951)	Mouse	IF (1:200)
**MVH/DDX4**	Abcam (ab13840)	Rabbit	IF (1:250)<br>IHC (1:400)
**TEX11**	Gifted by Chao Yu	Goat	IF(1:100)
**MZIP2 (SHOC1)**	Gifted by Chao Yu	Rabbit	IF (1:100)
**SYCP3**	Gifted by Chao Yu	Rat	IF (1:400)
**ac^4C**	Abcam (ab252215)	Rabbit	IF (1:200)<br>Dot-Blot (1:500)
**STRA8**	Abcam (ab49602)	Rabbit	IF (1:200)<br>WB (1:1000)
**γH2AX**	Millipore (#05-636)	Mouse	IF (1:200)
**γH2AX**	Cell Signaling (#9718S)	Rabbit	IF (1:400)<br>WB (1:1000)
**DMC1**	Proteintech (13714-1-AP)	Rabbit	IF (1:100)

**RAD51**	Abcam (ab176458)	Rabbit	IF (1:100)
**RPA2**	Abcam (ab76420)	Rabbit	IF (1:200)
**MLH1**	Proteintech (11697-1-AP)	Rabbit	IF (1:100)
**MSH4**	Abcam (ab58666)	Rabbit	IF: 1:50
**NAT10**	Abcam (ab194297)	Rabbit	IF (1:200)<br>IHC (1:400)<br>WB (1:1000)
**NAT10**	Proteintech (13365-1-AP)	Rabbit	IF (1:200)<br>WB (1:1000)
**β-Tubulin**	Trans （HC101-01）	Mouse	WB (1:1000)
**β-Actin**	Trans （HC201）	Mouse	WB (1:1000)
**GAPDH**	Trans （HC301）	Mouse	WB (1:1000)
**α-Tubulin**	Sigma (F2168)	Rabbit	WB (1:1000)

Supplementary Table S2. List of primer sequences related to experimental procedures

**Primer**	**Targeted**	**Application**	**Sequences (5'-3')**
Nat10-F	*Nat10*	Genotyping<br>WT: 186bp<br>fl: 290bp	5'-GGAACCATGAGTATTGTAGCCTGC-3'
Nat10-R			5'-CTATTGGCTGTGACTTCAGCAGAC-3'
Stra8-Cre-F	*Stra8-GFP KI-Cre*	Genotyping<br>WT: 702bp<br>KI: 400bp	5'-ACTCCAAGCACTGGGCAGAA-3'
Stra8-Cre-R1			5'-GCCACCATAGCAGCATCAAA-3'
Stra8-Cre-R2			5'-CGTTTACGTCGCCGTCCAG-3'
18S rRNA-F	18S rRNA	RT- PCR	5'- CGACGACCCATTCGAACGTCT -3'
18S rRNA-R			5'- CTCTCCGGAATCGAACCCTGA -3'
28S rRNA-F	28S rRNA	RT- PCR	5'-AGTCGGGTTGCTTGGGAATGC-3'
28S rRNA-R			5'-CCCTTACGGTACTTGTTGACT-3'
Actin-F1	*Actin*	RT- PCR	5'- GCTCTTTTCCAGCCTTCCTT-3'
Actin-R1			5'- GTACTTGCGCTCAGGAGGAG-3'
Gapdh-F:	*Gapdh*	RT- PCR	5'-CCCCAATGTGTCCGTCGTG-3'
Gapdh-R:			5'-TGCCTGCTTCACCACCTTCT-3'
